# A Novel Mechanism Inducing Genome Instability in Kaposi's Sarcoma-Associated Herpesvirus Infected Cells

**DOI:** 10.1371/journal.ppat.1004098

**Published:** 2014-05-01

**Authors:** Brian R. Jackson, Marko Noerenberg, Adrian Whitehouse

**Affiliations:** School of Molecular and Cellular Biology and Astbury Centre for Structural Molecular Biology, University of Leeds, Leeds, United Kingdom; Wistar Institute, United States of America

## Abstract

Kaposi's sarcoma-associated herpesvirus (KSHV) is an oncogenic herpesvirus associated with multiple AIDS-related malignancies. Like other herpesviruses, KSHV has a biphasic life cycle and both the lytic and latent phases are required for tumorigenesis. Evidence suggests that KSHV lytic replication can cause genome instability in KSHV-infected cells, although no mechanism has thus far been described. A surprising link has recently been suggested between mRNA export, genome instability and cancer development. Notably, aberrations in the cellular transcription and export complex (hTREX) proteins have been identified in high-grade tumours and these defects contribute to genome instability. We have previously shown that the lytically expressed KSHV ORF57 protein interacts with the complete hTREX complex; therefore, we investigated the possible intriguing link between ORF57, hTREX and KSHV-induced genome instability. Herein, we show that lytically active KSHV infected cells induce a DNA damage response and, importantly, we demonstrate directly that this is due to DNA strand breaks. Furthermore, we show that sequestration of the hTREX complex by the KSHV ORF57 protein leads to this double strand break response and significant DNA damage. Moreover, we describe a novel mechanism showing that the genetic instability observed is a consequence of R-loop formation. Importantly, the link between hTREX sequestration and DNA damage may be a common feature in herpesvirus infection, as a similar phenotype was observed with the herpes simplex virus 1 (HSV-1) ICP27 protein. Our data provide a model of R-loop induced DNA damage in KSHV infected cells and describes a novel system for studying genome instability caused by aberrant hTREX.

## Author Summary

The hallmarks of cancer comprise the essential elements that permit the formation and development of human tumours. Genome instability is an enabling characteristic that allows the progression of tumorigenesis through genetic mutation and therefore, understanding the molecular causes of genome instability in all cancers is essential for development of therapeutics. The Kaposi's sarcoma-associated herpesvirus (KSHV) is an important human pathogen that causes multiple AIDS-related cancers. Recent studies have shown that during KSHV infection, cells show an increase in a double-strand DNA break marker, signifying a severe form of genome instability. Herein, we show that KSHV infection does cause DNA strand breaks. Moreover, we describe a novel molecular mechanism for genome instability involving the KSHV ORF57 protein interacting with the mRNA export complex, hTREX. We demonstrate that over-expression of ORF57 results in the formation of RNA:DNA hybrids, or R-loops, that lead to an increase in genome instability. DNA strand breaks have been previously reported in herpes simplex, cytomegalovirus and Epstein-Barr virus infected cells. Therefore, as this work describes for the first time the mechanism of R-loop induced genome instability involving a conserved herpesvirus protein, it may have far-reaching implications for other viral RNA export factors.

## Introduction

Genome instability, an enabling characteristic of the hallmarks of cancer, has long been established as a major contributing factor to cancer formation and progression [1], [2]. However, our understanding of the underlying molecular causes is still in its relative infancy. Contributing factors of genome instability are wide ranging and incorporate those from exogenous sources, such as ionising radiation, endogenous sources such as reactive oxygen species (ROS) and reactive nitrogen species (RNS), as well as mutations incorporated into the genome during cell replication, including DNA replication errors and error prone DNA repair [3], [4]. Cells have evolved to deal with this onslaught of damage through several DNA repair pathways, each specific to certain types of damage [4]. The most severe types of DNA damage result in double strand breaks (DSB) that can be repaired primarily through error-free homologous recombination (HR) [5], [6], or error-prone non-homologous end-joining (NHEJ) [6]. DSBs are closely associated with cancer progression and can include severe chromosome pulverisation and chromothripsis [7]–[9] leading to major chromosome rearrangements, as well as smaller mutations. As such, DSBs are known to be an integral part of many cancers, for example, breast cancers, Burkitt's lymphoma, and multiple leukaemia's [10]–[12].

The Kaposi's sarcoma-associated herpesvirus (KSHV) is an important oncogenic virus associated with multiple AIDS-associated malignancies including Kaposi's sarcoma (KS), primary effusion lymphoma (PEL) and multicentric Castleman's disease (MCD) [13], [14]. Like all herpesviruses, KSHV has a bi-phasic lifecycle incorporating latency and lytic replication [15]. During latency the virus expresses a small subset of genes that allows it to persist in the host cell, while reactivation to the lytic cycle results in expression of the full viral genome and production of infectious virus progeny. KSHV differs from the other oncogenic herpesviruses as both the latent and lytic cycles are required for tumorigenesis [13], [16]. Interestingly, KSHV has recently been shown to cause DNA damage in infected cells [17]. Specifically, lytic infection of cells has been shown to directly induce DNA double-strand breaks, a severe form of genome instability [17]. Moreover, several reports have identified chromosomal instability in KSHV infected cells, which have been suggested to contribute to the neoplastic process of KSHV [18], [19]. Specifically, loss of chromosomes 14 and 21. and non-random translocations and deletions in chromosome 3 [19]–[21]. Furthermore, loss of the Y chromosome in early tumour stages, recurrent gains in chromosome 11 and further chromosomal stages during late KSHV-associated tumour stages have been reported [22]–[24]. Importantly however, there is currently no known mechanism that describes how KSHV lytic replication can cause DNA double-strand breaks.

Recent evidence has suggested there may be a surprising link between mRNA export, genome instability, and cancer development [25]. The Transcription and Export complex (TREX) [26], has multiple roles throughout mRNA processing including recruitment of the nuclear export receptor, TAP, to initiate efficient bulk mRNA export, as well as stabilisation of mRNA during transcription [27]. Human TREX (hTREX) comprises the DEAD-box helicase UAP56, the export adapters Aly and UIF, the recently discovered CIP29 , PDIP3 , Chtop and ZC11A proteins, and the multi-protein THO complex (summarised in Schumann *et al* [28]). Aberrations that affect hTREX protein expression and function have been implicated in human cancer [25], [29]. For example, the export adapter protein Aly, which functions to recruit the hTREX complex to the mRNA, as well as the THO component THOC1, are known to be deregulated in multiple cancers [25], [29], [30]. Strikingly, there appears to be disparity not only between different cancer types, but also between low- and high-grade tumours. THOC1 is overexpressed in ovarian, colon and lung cancers, but there is a loss of expression in testicular and skin cancer [30]. Moreover, while Aly is highly expressed in several low-grade lesions, it is undetectable in multiple high-grade tumours of the colon, stomach, thyroid, testis and skin [30]. Furthermore, UAP56 has been identified as having a possible role in causing chromosome instability during mitosis, where knock-down of UAP56 leads to premature sister chromatid separation [31]. Together, these observations highlight the importance of the hTREX complex, not only in exporting mRNAs from the nucleus, but also in maintaining genome integrity during transcription. Thus, aberrations in components of hTREX can have devastating consequences on genome stability [31]. Importantly, a body of work, primarily performed in yeast, has highlighted a potential mechanism for how defects in mRNA export may contribute to genome instability [32]. Yeast THO mutants show impaired transcription elongation and RNA export defects, as well as a high level of transcription-associated recombination. It is thought that an absence of THO at the 5′ end of a nascent mRNA during transcription leads to the loss of mRNA stability and the formation of abnormal RNA:DNA hybrids known as R-loops. These R-loops may form as the newly transcribed mRNA anneals to the template strand of the DNA, and although a mechanism has been proposed [33] our understanding of the processes involved is still lacking.

The KSHV open reading frame 57 (ORF57) protein is a multi-functional protein that, like its homologues in other herpesviruses, facilitates all stages of viral mRNA processing throughout lytic replication [28], [34]–[37]. Through an interaction with the KSHV transactivator protein, Rta, ORF57 has been shown to function co-transcriptionally promoting expression of KSHV genes [38], [39]. ORF57 is also known to enhance the splicing of several viral transcripts [40]. Moreover, through an interaction with the cellular protein PYM, ORF57 is able to facilitate the recruitment of the cellular pre-initiation complex and enhance the pioneer round of viral translation [41], [42]. The majority of work on ORF57 has focused on its roles in RNA stability and mRNA export [43]. Several recent studies have shown the importance of ORF57 in stabilising the KSHV PAN RNA [44]–[46]. Importantly, ORF57 also acts as a viral mRNA export factor and recruits the entire hTREX complex to viral mRNA through a direct interaction with the export adapter proteins Aly and UIF [47]–[49]. The interaction with these two separate cellular export adapter proteins allows for redundancy in the ORF57-mediated viral mRNA export. Significantly, ORF57 has been shown to be essential for KSHV replication [50], and ORF57-mediated recruitment of hTREX to viral mRNA is essential for efficient KSHV lytic replication [47].

The link between mRNA export and genome instability is intriguing. We therefore set out to investigate whether the known interaction between ORF57 and hTREX could have implications for genome instability in KSHV lytically infected cells. Herein, we demonstrate that sequestration of hTREX by the KSHV ORF57 protein is sufficient to induce genome instability, due to a consequence of the formation of R-loops. This work highlights the importance of viral models for our understanding of cellular processes, and also demonstrates and confirms a novel link between mRNA export and genome instability, a major driving force behind tumorigenesis. Moreover, it describes a novel mechanism to account for the DNA damage observed in lytically active KSHV infected cells.

## Results

### KSHV lytic expression induces a DSB response and causes DNA strand breaks

**Figure 1:**
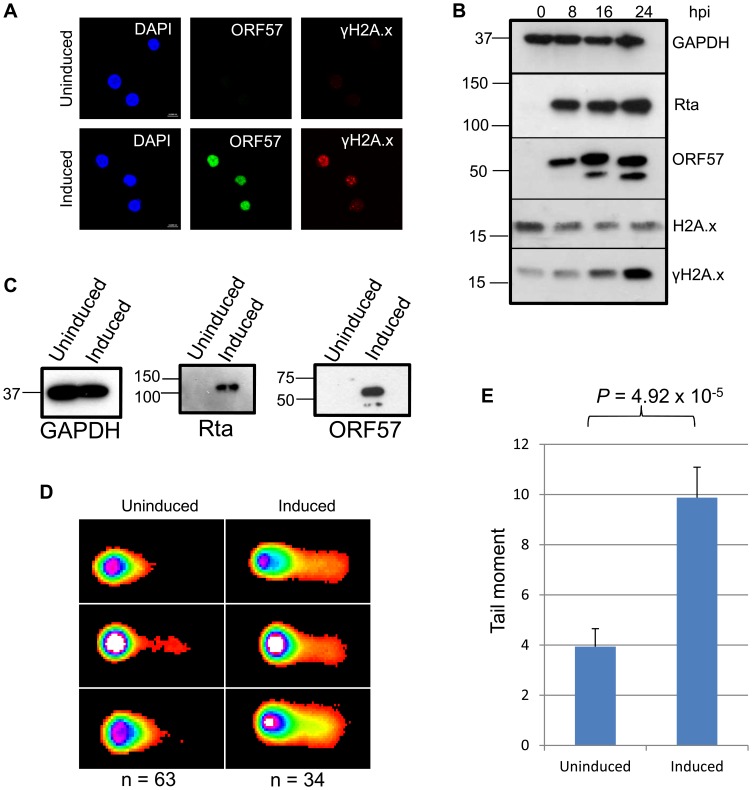
KSHV lytic reactivation induces the double strand break response, and the formation of DNA breaks. (**A**), TREx BCBL Rta cells remained unreactivated or were reactivated for 24 hours and fixed for immunofluorescence. Cells were then stained using a monoclonal antibody to ORF57 to show reactivation and a monoclonal antibody to γH2A.x to show sites of DNA damage. (**B**) A time-course of protein expression in reactivated TREx BCBL Rta cells over 0, 8, 16 and 24 hours post induction. A monoclonal antibody to GAPDH was used to show equal loading, and monoclonal antibodies to Myc for Rta and to ORF57 were used to show KSHV reactivation. To demonstrate DNA damage, a polyclonal antibody was used to show total levels of H2A.x and a monoclonal antibody for the phosphorylated form, γH2A.x. (**C**) For comet assays, TREx BCBL Rta cells were reactivated for 24 hours and reactivation assessed by western blot using monoclonal antibodies to Myc for Rta and to ORF57. A monoclonal antibody to GAPDH was also used to show equal loading. (**D**). Comet assays were performed and scored using CometScore and tail moments calculated (**E**); n- and *P*-values are represented in the figure and error bars show the standard error from the mean.

KSHV has been shown to induce genome instability either through chromosome instability [18] or, more recently, through the observation that lytic KSHV infection in BCBL-1 cells reactivated with TPA and sodium butyrate induces the phosphorylation of the double strand break marker, γH2A.x [17]. To confirm this data in a well characterised KSHV infected cell line, we utilised the Tet-regulated expression system T-REx with the TREx BCBL1-Rta cell line [51]; a KSHV latently infected cell line containing a Myc-tagged version of the viral transcriptional activator, Rta, under the control of a doxycycline-inducible promoter. We first used confocal fluorescence microscopy to assess the phosphorylation of the H2A histone variant, H2A.x, as a marker for DNA strand breaks [52]. After 24 hours, doxycycline induced TREx BCBL1-Rta cells showed significant levels of the phosphorylated form of H2A.x, γH2A.x compared to uninduced cells (Figure 1A). Furthermore, we induced TREx BCBL1-Rta cells over a 24 hour time-course and analysed cell lysates by western blotting. The KSHV early gene product, ORF57, can be seen at 8 hours post-induction with increasing expression at 16 and 24 hours (Figure 1B). ORF57 is observed as a double band to differing degrees in different cell lines due to a known caspase-7 cleavage event [53]. Interestingly, γH2A.x increases through the same time-course concurrent with ORF57, while total levels of H2A.x are slightly decreased. It is important to note that an interesting recent study highlighted the importance of γH2A.x for KSHV episome persistence [54]. This study demonstrated that H2A.x is phosphorylated during KSHV latency, confirmed by our western blot analysis (Figure 1B), but our data convincingly show that this level is dramatically increased during the KSHV lytic replication cycle.

To analyse DNA damage directly in KSHV lytically infected cells we performed comet assays on uninduced versus induced TREx BCBL1-Rta cells. Cells were first induced for 24 hours allowing sufficient ORF57 expression (Figure 1C) before alkaline comet assays were performed to assess the total level of single and double strand DNA breaks (Figure 1D and 1E). Tail moments were scored giving values of 3.93 for uninduced TREx BCBL1-Rta cells versus 9.87 for induced cells indicating a significant level of DNA strand breaks. Data were analysed using an unpaired 1-tailed T-test to verify the statistical significance (*P* = 4.92×10^−5^). Together, these data show the relevance of KSHV as a model of genome instability and, using an inducible KSHV system, confirm previous observations that KSHV lytic replication induces DNA double-strand breaks.

### Over-expression of KSHV ORF57 leads to genome instability and provides a model for hTREX sequestration

To assess the effect of ORF57 expression on the cellular proteome, and in particular on DNA repair pathways, we undertook a stable isotope labelling by amino acids in cell culture (SILAC) based quantitative proteomics approach [55], [56]. The Flp-In T-REx-293 system was used to create a stable KSHV ORF57 inducible cell line, iORF57-293, with ORF57 under the control of a tetracycline/doxycycline-inducible promoter. Uninduced cells were grown in heavy isotope labelled DMEM, R6K4, while the sample to be induced was grown in label-free DMEM, R0K0, for 6 passages to allow incorporation of the respective isotopes. Cells grown in DMEM R0K0 were then induced for 24 hours to allow for expression of ORF57 before being fractionated into cytoplasmic, nuclear and nucleolar fractions to reduce sample complexity. The quality of the cellular fractions was confirmed using specific markers to cellular proteins (Figure S1). Protein samples were separated by SDS-PAGE and stained with colloidal blue stain. Each protein gel lane was excised in 10 fragments, digested with trypsin and analysed by LC-MS mass spectrometry (LTQ-Orbitrap Velos, service provided by the University of Dundee). Quantification of peptide changes was performed using MaxQuant [57], [58] and expressed as a fold change of endogenous proteins in induced cells compared to uninduced cells. A 2.0-fold cutoff was chosen as a basis for investigating potential proteome changes between data sets. Bioinformatical analysis was performed using IPA analysis software (Ingenuity systems) and multiple proteins were found to be enriched upon ORF57 expression, including many associated with DNA repair (Table S1). Of particular interest were proteins involved in the error-prone double-strand repair pathway, NHEJ. Analysis of cellular pathways highlighted that the majority of the proteins involved in this repair pathway are enriched upon ORF57 expression (Table 1). Indeed, Ku70 and Ku80 are enriched by 3.2-fold and 2.9-fold, respectively, DNA-PK by 2.1-fold and Rad50 by 2.4-fold. This enrichment suggests a possible activation of DSB repair upon ORF57 expression.

**Table 1:** SILAC analysis demonstrates the induction of double strand breaks in ORF57 expressing cells.

Protein	UniProt number	Fold increase	Number of peptide hits
**RAD50**	Q92878	2.44	49
**DNA-PK**	P78527	2.13	228
**KU70**	P12956	3.23	38
**KU80**	P13010	2.90	42
**PARP1**	P09874	2.83	65
**XRCC1**	P18887	2.68	11
**DNA ligase 3**	P49916	2.34	22
**MRE11**	B3KTC7	2.77	14

Fold-increases of proteins involved in NHEJ upon ORF57 expression are presented along with the number of peptide hits for each protein. UniProt numbers are shown for reference.

**Figure 2:**
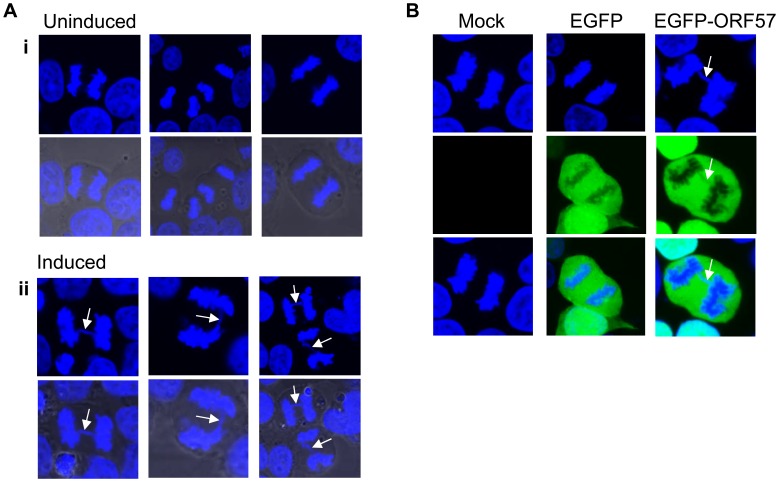
Chromosome instability in ORF57 expressing cells. (**A**) iORF57 293 cells remained uninduced or induced for 24 hours, then fixed on coverslips and DNA-stained with DAPI. Confocal microscopy was used to identify and image mitotic cells. Three representative images are shown for uninduced and induced cells and merged images include the phase contrast image. Chromosome lagging in the induced samples is highlighted by white arrows. (**B**) HEK 293T cells mock transfected or transfected with EGFP or EGFP-ORF57 were fixed onto coverslips and stained with DAPI for imaging of mitotic cells. Chromosome lagging in the EGFP-ORF57 expressing cell is highlighted by a white arrow.

To demonstrate that ORF57 expression alone is sufficient to induce chromosome instability, as has previously been reported for KSHV infection [18], we undertook confocal fluorescence microscopy of mitotic cells comparing uninduced versus induced ORF57-expressing cells to look for characteristic markers of genome instability. In the first instance we looked for the presence or absence of chromosomal anomalies in the form of lagging chromosomes during mitosis where chromosomes do not separate correctly into daughter cells, an aberration that has been shown to have links to defective hTREX [31]. This can lead to the formation of micronuclei, a common occurrence in many tumour cells [59]. iORF57-293 induced cells showed numerous chromosome abnormalities in the form of chromosome lagging (Figure 2A) compared to uninduced cells, implying that ORF57 expression alone has a significant effect on genome maintenance. This observation was confirmed in HEK 293T cells either mock transfected, transfected with an EGFP or an EGFP tagged form of ORF57, EGFP-ORF57. Only the cells expressing EGFP-ORF57 were observed to contain chromosome lagging (Figure 2B). Of the total cells examined, only 1 out of 22 showed chromosome lagging in cells not expressing ORF57 compared to 6 out of 14 in cells expressing ORF57. The importance of this observation is still to be fully characterised, but it demonstrates the ability of ORF57 to induce genome instability in cells. Moreover, there is an established link between hTREX siRNA knockout and mitotic anomalies [31] that suggests interfering with hTREX function, for example with the over-expression of ORF57, could lead to the formation of mitotic anomalies such as chromosome lagging. These data together suggest that ORF57 expression is sufficient to induce a DSB response and increase the levels of chromosome instability in cells.

### KSHV ORF57 expression alone is sufficient to elicit a DSB response

**Figure 3:**
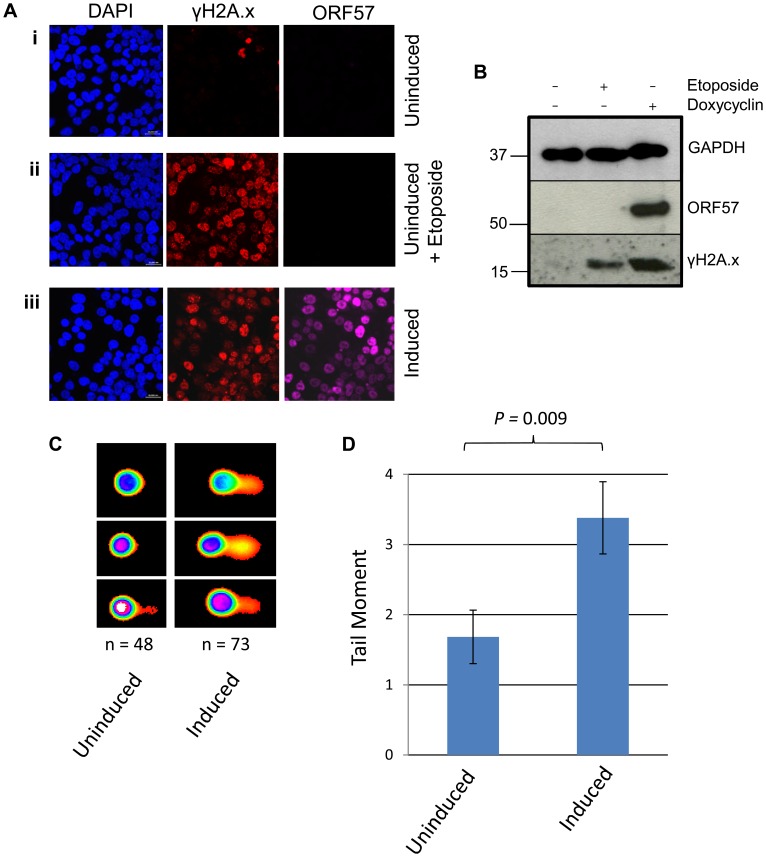
ORF57 expression induces the DNA damage response through phosphorylation of H2A.x. (**A**) iORF57 293 cells were either uninduced, treated with 50 µM etoposide for 30 minutes to induce DSBs, or induced to express ORF57, fixed on coverslips and visualised by confocal microscopy. A polyclonal antibody against Flag was used to detect ORF57 and a monoclonal antibody against γH2A.x was used to demonstrate cells with DSBs. (**B**) iORF57 293 cells were either uninduced, treated with 50 µM etoposide for 30 minutes, or induced for 48 hours. Cell lysates were collected and western blots performed to analyse protein levels. A monoclonal antibody to GAPDH was used to show equal loading, a polyclonal antibody to Flag to show ORF57 expression and a monoclonal antibody to γH2A.x to demonstrate DSBs. (**C**) iORF57 293 cells were either uninduced or induced for 48 hours and alkaline comet assays were performed. (**D**) Comets were scored using CometScore and tail moments calculated. n- and *P*-values are represented in the figure and error bars show the standard error from the mean.

To further confirm that ORF57 expression alone is required for a DSB response we performed confocal fluorescence microscopy on uninduced versus induced iORF57-293 cells and assayed γH2A.x levels (Figure 3A). Uninduced cells showed minimal γH2A.x staining, as would be expected of a healthy cell population. As a positive control the topoisomerase II inhibitor etoposide was used to treat the cells for 30 minutes at a concentration of 50 µM leading to increased levels of γH2A.x. Importantly, ORF57 induction also led to an increased level of γH2A.x, confirming that ORF57 expression promotes the DSB response. Moreover, western blot analysis of iORF57-293 cell lysates shows an increase in γH2A.x upon ORF57 expression when compared to uninduced cells (Figure 3B). Additional controls confirmed that the parental cell line, 293 Flp-In, shows low levels of γH2A.x (Figure S2). Further to this we performed alkaline comet assays on iORF57 293 cells that had been left uninduced or induced to express ORF57 for 16 hours (Figure 3C and 3D). Uninduced cells had a tail moment of 1.68 compared to 3.38 for induced cells (*P* = 0.009) demonstrating that ORF57 expression can lead directly to DNA strand breaks.

**Figure 4:**
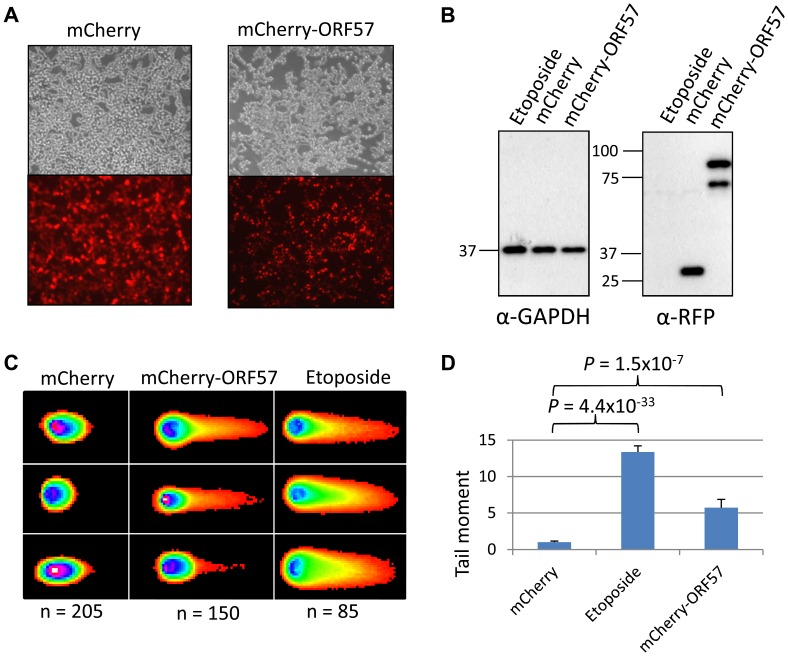
ORF57 induces double strand breaks in a neutral comet assay. Neutral comet assays were performed to assess double-strand breaks. (**A**) Cells were either treated with 50 µM etoposide for 15 minutes as a positive control, transfected with mCherry, or transfected with mCherry-ORF57 and analysed by fluorescence microscopy to assess transfection efficiency. (**B**) Protein expression was assessed by western blot using a monoclonal antibody to GAPDH as a loading control and polyclonal antibody to RFP to detect mCherry and mCherry-ORF57 expression. (**C**) Comets were performed and scored using CometScore and (**D**) tail moments calculated. N- and p- values are shown and error bars represent the standard error from the mean.

In addition, we performed neutral comet assays to demonstrate the presence of double strand DNA breaks in an over-expression system using mCherry-ORF57, mCherry as a negative control and etoposide treatment as a positive control (50 µM for 15 mintues) (Figure 4). As the assay is reliant on transfection, the transfection efficiency was confirmed by immunofluorescence microscopy, and protein expression levels determined by western blot (Figure 4A and 4B). mCherry-ORF57 expressing cells showed significant DNA damage with a tail moment of 7.59 compared to 1.32 for mCherry expressing cells (*P* = 1.53×10^−7^) (Figure 4C and 4D). These data strongly support the conclusion that ORF57 expression leads to DNA damage.

Furthermore, western blot analysis was performed on the total protein levels of ORF57 and Aly. iORF57-293 cells either left uninduced or induced for 16 hours to express ORF57, 293T cells either mock transfected or transfected for 24 hours with EGFP-ORF57 and a HEK 293T based cell line containing the entire KSHV genome, termed 293T rKSHV.219 [60], that was reactivated with 20 ng/ml TPA and 1.5 mM sodium butyrate for 36 hours showed that the relative levels of Aly remain relatively unchanged upon ORF57 expression (Figure S3). There is a slight increase in Aly expression in iORF57-293 cells upon induction of ORF57 which could be explained by a compensatory effect by the cells due to the sequestration of hTREX away from cellular transcription. Moreover, we endeavoured to investigate the effect that ORF57 over-expression has on cellular nuclear export of polyadanylated mRNA as it seems likely that ORF57 recruitment of hTREX would mirror a hTREX knockdown (Figure S4). To this end we performed fluorescence *in situ* hybridisation (FISH) experiments on 293T cells either mock transfected, transfected with EGFP or transfected with EGFP-ORF57. Importantly, no effect was observed on polyadenylated mRNA in mock transfected or EGFP transfected cells. However, EGFP-ORF57 over-expression had a marked impact on the subcellular localisation of polyadenlyated mRNA with a large proportion retained in the nucleus. Interestingly, the retained polyadenylated mRNA does not co-localise completely with ORF57, suggesting that it is not ORF57 directly that is retaining the cellular mRNA. This data shows convincingly that ORF57 binding to hTREX mimics a hTREX knockdown and causes a block to bulk cellular mRNA export.

### Sequestration of hTREX by the KSHV ORF57 protein leads to R-loop formation and genome instability

**Figure 5:**
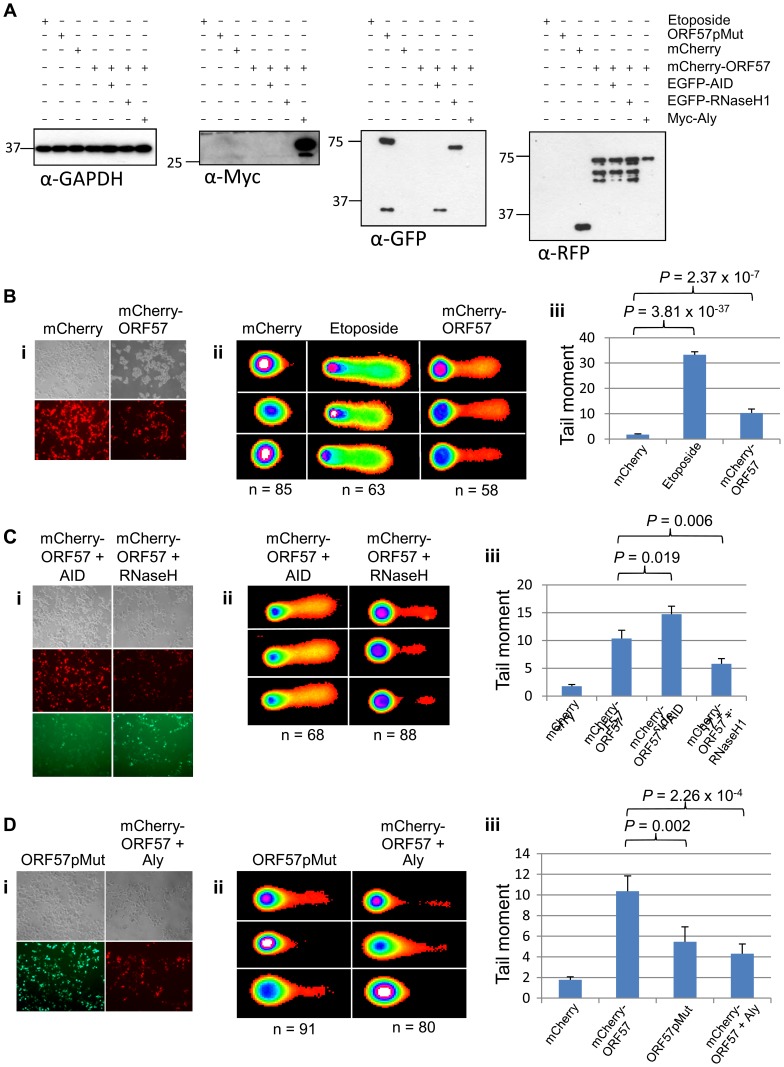
ORF57-induced double strand breaks are the result of R-loop formation and sequestration of hTREX. (**A**) Alkaline comet assays were performed on HEK 293T cells and protein levels for each assay were assessed by western blot using a monoclonal antibody to GAPDH as a loading control; monoclonal antibody to Myc for Myc-Aly; monoclonal antibody to GFP for ORF57pMut, EGFP-RNaseH1 and EGFP-AID; polyclonal antibody to RFP for mCherry and mCherry-ORF57. (**B**) Cells were transfected with mCherry or mCherry-ORF57, or treated with 50 µM etoposide for 30 minutes as a positive control, visualised by fluorescence microscopy to confirm transfection efficiency (**i**); comet assays performed and scored using CometScore (**ii**); and tail moments calculated (**iii**). (**C**) To demonstrate R-loop formation, cells were co-transfected with mCherry-ORF57 and either pMSCVgfp::AID or pEGFP-RNH, visualised by fluorescence microscopy to confirm transfection efficiency (**i**); comet assays performed and scored using CometScore (**ii**); and tail moments calculated (**iii**). (**D**)To demonstrate sequestration of hTREX by ORF57 as the cause of R-loop formation, cells were either transfected with pEGFP-57pmut or co-transfected with mCherry-ORF57 and Myc-Aly visualised by fluorescence microscopy to confirm transfection efficiency (**i**); comet assays performed and scored using CometScore (**ii**); and tail moments calculated (**iii**). n- and *P*-values are represented for all data in the figure and error bars show the standard error from the mean.

We have previously shown that ORF57 recruits the entire hTREX complex [4], [47], [61]. Taking into account the link between hTREX aberrations and genome instability, we hypothesised that the DSB response observed upon ORF57 expression could be due to the interaction between ORF57 and hTREX. To test this hypothesis we undertook a series of comet assays in HEK 293T cells expressing ORF57. Initially, we tested whether ORF57 expression alone led to an increase in single and double strand breaks using alkaline comet assays. Cells were either transfected with a construct expressing mCherry, or an mCherry tagged ORF57 (mCherry-ORF57), as well as untransfected cells treated with etoposide (50 µM for 15 minutes) as a positive control. Western blot analysis shows exogenous protein expression (Figure 5A) and fluorescence microscopy images are provided to show the high level of transfection efficiency (Figure 5B(i)). Alkaline comet assays were performed to determine the level of total single and double strand breaks (Figure 5B). Cells transfected with mCherry showed minimal levels of DNA damage with a tail moment of 1.78, compared to 33.27 for etoposide treated cells (*P* = 3.81×10^−37^, unpaired 1-tailed T-tests were used for this and all subsequent statistical analyses). Notably, cells expressing mCherry-ORF57 also showed significant DNA damage with a tail moment of 10.35 (*P* = 2.37×10^−7^ when compared to mCherry transfected cells). These findings are consistent with our previous observations that ORF57 expression induces a DSB response and causes DNA damage and genome instability.

The loss of hTREX function has previously been shown to lead to the formation of R-loops in both yeast and siRNA knockdown in human cells [33], [62]. We hypothesise that ORF57 sequestration of hTREX away from cellular transcription to sites of viral transcription could lead to a loss of cellular mRNA stability and the formation of R-loops. To determine this, we utilised a technique shown previously to confirm the presence of R-loops that involves the over-expression of RNaseH and activation-induced cytidine deaminase (AID). The principle being that an R-loop consists of an RNA:DNA hybrid and that overexpression of an RNaseH enzyme in a system containing R-loops will lead to the degradation of the RNA component allowing resolution of the R-loop and a reduction in DNA damage [63]. Alternatively, overexpression of AID can lead to a state of hypermutation [64] whereby the single-stranded DNA present in an R-loop is subjected to increased deamination that would lead to an increase in the level of observed DNA damage. HEK 293T cells were again transfected with mCherry-ORF57 along with either EGFP-RNaseH1 or pMSCVgfp::AID (Figure 5C(i)). The cells expressing mCherry-ORF57 and pMSCVgfp::AID showed significant increase in DNA damage with a tail moment of 14.72 (*P* = 2.72×10^−13^ compared to mCherry expressing cells and *P* = 0.019 compared to mCherry-ORF57 expressing cells) (Figure 5C). This significant increase demonstrates that in cells expressing ORF57 there is single stranded DNA present, indicative of R-loops. Moreover, cells expressing mCherry-ORF57 and EGFP-RNaseH1 had a tail moment of 5.81 and showed a significant decrease in the level of DNA damage when compared to mCherry-ORF57 expression alone (*P* = 0.006) (Figure 5C), indicating the presence of RNA:DNA hybrids. Importantly, together these data confirm that ORF57 induces the formation of R-loops.

Finally, to confirm that R-loop formation in the presence of ORF57 is a consequence of ORF57 sequestering hTREX away from cellular transcription and to sites of viral transcription, we first transfected HEK 293T cells with an ORF57 mutant, ORF57pmut, which has previously been shown to be unable to interact with Aly and recruit the remainder of the hTREX complex (Figure 5D(i)) [49]. If indeed ORF57 does induce R-loops and the subsequent genome instability is due to sequestration of hTREX this mutant should show a reduction in DNA damage. Expression of ORF57pmut produced a tail moment of 5.46, significantly lower than expression of mCherry-ORF57 (*P* = 0.002) (Figure 5D). It should be noted that although this mutant is unable to recruit hTREX via Aly the inherent redundancy within hTREX means that UIF could be recruited, albeit at a slightly lower level. This could explain the observation that there is still a level of DNA damage in ORF57pmut expressing cells. To further investigate this sequestration mechanism, we co-transfected HEK 293T cells with constructs expressing mCherry-ORF57 as well as a Myc-tagged Aly construct (Figure 5D(i)). Myc-Aly in this system should function as a molecular sponge and prevent ORF57 from sequestering all of the endogenous hTREX proteins, thereby reducing the level of DNA damage observed. Strikingly, Myc-Aly overexpression did reduce the tail moment to 4.31 (*P* = 2.26×10^−4^). Further controls were performed on cells transfected with pMSCVgfp::AID alone, or transfected with EGFP-RNaseH1 alone, and show no significant increase in tail moment compared to mock transfected cells (Figure S5). Taken together, these results suggest that sequestration of hTREX by ORF57 results in the observed genome instability due to of R-loop formation.

### DNA double strand breaks in KSHV infected cells can be attributed to R-loop formation and hTREX sequestration

**Figure 6:**
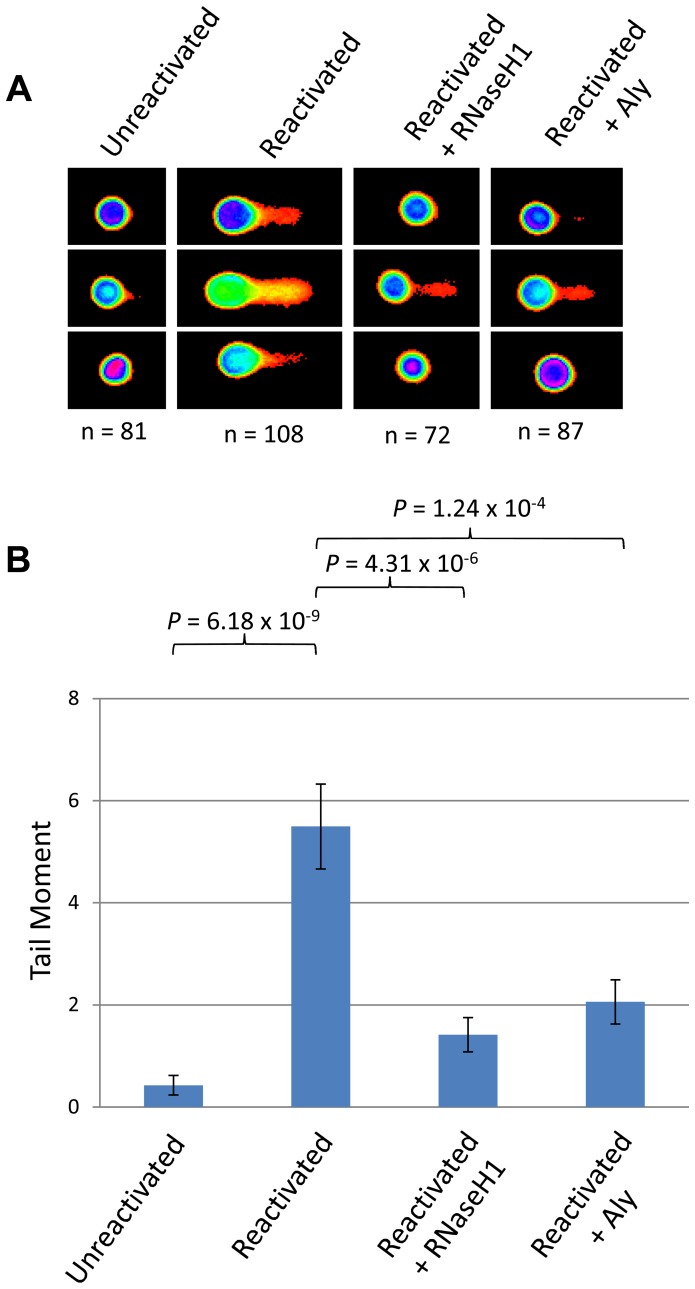
Lytically replicating KSHV cells demonstrate the presence of R-loops. (**A**) 293T rKSHV.219 cells were left either unreactivated, or reactivated for 36 hours and transfected with Myc-Aly or EGFP-RNaseH1 and alkaline comet assays were performed. (**B**) Comet tails were scored using CometScore and n- and *P*-values are represented for all data and error bars show the standard error from the mean.

To examine whether our hypothesis of hTREX sequestration by ORF57 away from cellular transcription to sites of viral transcription leads to R-loop formation during KSHV infection we utilised the HEK 293T based cell line containing the entire KSHV genome, 293T rKSHV.219. We performed a series of alkaline comet assay experiments to determine the levels of DNA strand breaks comparing KSHV latent and reactivated cells (Figure 6). Unreactivated cells showed a tail moment of 0.43, whilst after being reactivated using 20 ng/ml TPA and 1.5 mM sodium butyrate for 36 hours cells showed a significant increase in the tail moment to 5.49 (*P* = 6.18×10^−9^) demonstrating a level of DNA strand breaks. This confirms our previous observations in TREx BCBL-Rta cells showing that KSHV lytic replication directly causes DNA damage. To determine whether the observed DNA damage could be attributed, at least in part, to R-loop formation we again utilised a construct expressing EGFP-RNaseH1 and transfected it into the 293T rKSHV.219 cells 8 hours prior to reactivation. Importantly, there was a significant decrease in the tail moment when compared to the reactivated cells without EGFP-RNaseH1 over-expression down to 1.42 (*P* = 4.31×10^−6^). This demonstrates that the DNA strand breaks in KSHV lytically replicating cells are as a result of the formation of R-loops. Moreover, to demonstrate that the R-loop formation is as a result of ORF57 sequestering hTREX away from cellular transcription to sites of viral transcription we transfected Myc-Aly into the 293T rKSHV.219 cells. As was observed in the ORF57 over-expression system this again dramatically and significantly reduced the tail moment to 2.06 (*P* = 1.24×10^−4^ when compared to reactivated 293T rKSHV.219 cells) indicating that hTREX sequestration by ORF57 is the cause of R-loop formation and DNA strand breaks in lytically active KSHV infected cells.

### The ORF57 homologue of herpes simplex virus type-1, ICP27, shows a similar effect on genome instability

**Figure 7:**
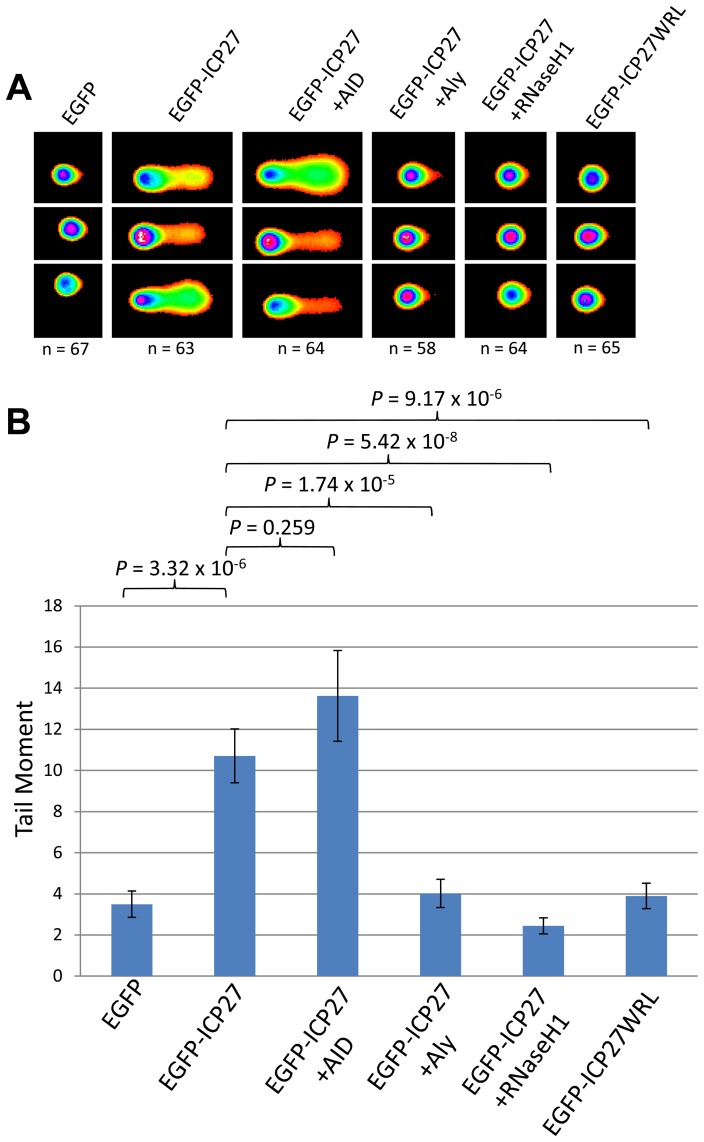
HSV-1 ICP27 induces R-loop mediated DNA damage due to hTREX recruitment. (**A**) HEK 293T cells were transfected with EGFP, EGFP-ICP27, EGFP-ICP27WRL or a dual transfections of EGFP-ICP27 along with Myc-Aly, EGFP-RNaseH1 or pMSCVgfp::AID for 24 hours alkaline comet assays were performed. (**B**) Comet tails were scored using CometScore and n- and *P*-values are represented for all data and error bars show the standard error from the mean.

It follows that if ORF57 is capable of recruiting hTREX in such a way as to mimic hTREX knockdown leading to R-loops and genome instability, that other herpesvirus homologues of ORF57 that interact with hTREX could function in a similar way. We therefore decided to investigate whether the ORF57 homologue of HSV-1, ICP27, was sufficient to elicit a similar response. We chose ICP27 as a DNA damage response has been observed in HSV-1 infected cells and ICP27 is known to interact with hTREX via a direct interaction with Aly [65], [66]. Moreover, mutant proteins are available which abolish the ICP27-Aly interaction [67]. Alkaline comet assays were again performed on 293T cells transfected with EGFP-ICP27 (Figure 7). Cells expressing EGFP-ICP27 showed a tail moment of 10.71 compared to 3.50 for EGFP transfected cells (*P* = 3.32×10^−6^), demonstrating that ICP27 is indeed sufficient to induce a significant amount of DNA strand breaks. To assess whether the observed DNA damage is a consequence of R-loop formation cells were co-transfected with EGFP-ICP27 along with either pMSCVgfp::AID or EGFP-RNaseH1. AID expression led to an increase in the tail moment to 13.62, although not significant (*P* = 0.259) whereas RHaseN1 expression significantly decreased the tail moment to 2.45 (*P* = 5.42×10^−8^), indicating that, similar to KSHV ORF57, HSV-1 ICP27 causes DNA damage via R-loop formation. To further characterise the mechanism we either co-transfected EGFP-ICP27 along with Myc-Aly or transfected a mutant of ICP27 which fails to interact with Aly, known as EGFP-ICP27WRL [65], [67]. Again, as expected, both showed reduced tail moments when compared to EGFP-ICP27 alone (4.03 and 3.90 respectively, with *P*-values of 1.74×10^−5^ and 9.17×10^−6^) suggesting that the R-loop formation is as a result of ICP27 recruiting hTREX away from cellular transcription.

## Discussion

**Figure 8:**
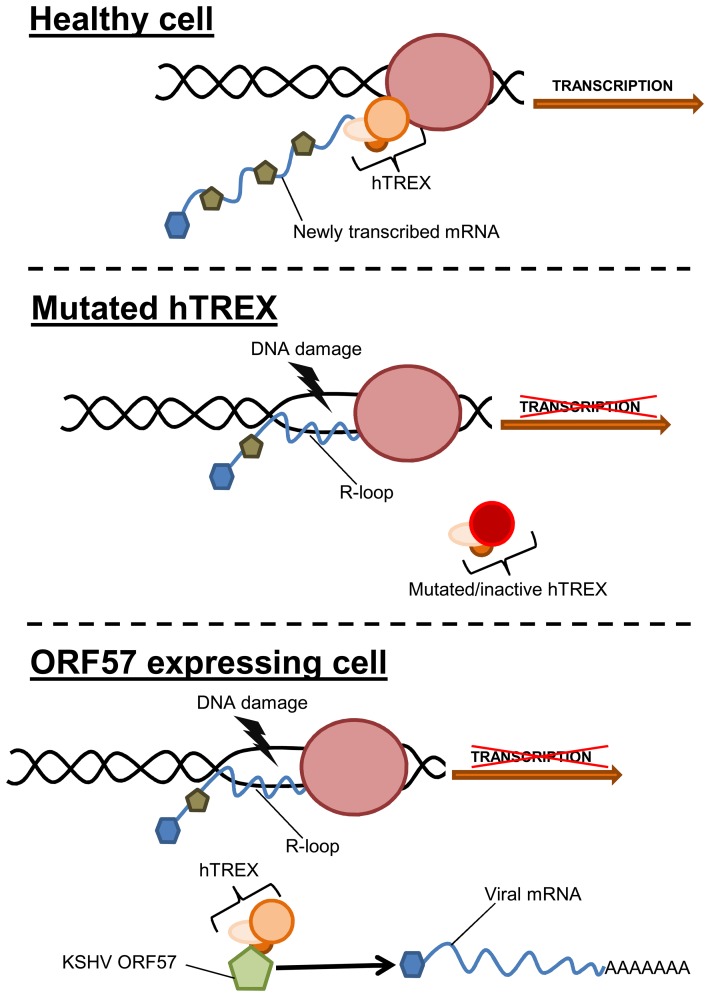
Model of how sequestration of hTREX by ORF57 leads to R-loops and genome instability. In a healthy cell, components of hTREX are recruited to cellular pre-mRNA during the inter-linked processes of transcription and splicing. These components then act to stabilise the newly transcribed mRNA. In situations when hTREX is rendered non-functional through mutation or siRNA, the newly transcribed mRNA can become unstable and anneal to the template strand of DNA forming R-loops and leading to an increase in DNA strand breaks. During KSHV infection or exogenous ORF57 expression, ORF57 recruits the hTREX complex, essentially replicating a system of mutated hTREX and leading to an increase in genome instability.

The role of hTREX in genome stability and cancer is an important emerging field [25], [29]. As we have previously shown, the KSHV ORF57 protein interacts directly with hTREX [47], [68]. Moreover, KSHV is known to induce DNA double-strand breaks during lytic replication, although no mechanism has been previously described [17], [49]. Therefore, we set out to investigate what effect sequestration of hTREX by KSHV ORF57 would have upon R-loop formation and genome instability, and whether this would describe a mechanism by which KSHV could induce DNA strand-breaks. The data presented here convincingly show that using the KSHV mRNA export factor, ORF57, we can accurately replicate a system deficient in hTREX and that this lack of hTREX does lead to R-loop-mediated genome instability in KSHV infected cells. Our model proposes that aberrant hTREX levels, sequestered by ORF57, leads to the formation of R-loops, which in turn increases the number of DNA DSBs and the rate of mutation. This model explains for the first time a possible novel mechanism of lytic KSHV induced DNA double-strand breaks (Figure 8). We suggest that this model could also describe a possible driving force behind KSHV tumorigenesis as several studies have shown significant defects in proteins involved in mRNA processing in numerous cancers [25], [29], [30]. Importantly, the observed decrease in DNA damage in 293T rKSHV.219 cells when either Aly or RNaseH1 are over-expressed demonstrates that this model is viable in the context of a KSHV infection. The complex nature of lytic replication in KSHV makes it difficult to confirm that all observed DNA damage is as a result of ORF57-induced R-loops as other studies have shown a DNA damage response caused by other KSHV proteins, for example v-cyclin [69] and LANA [54], [70]. However, our data present compelling evidence that R-loop formation as a result of ORF57 sequestration of hTREX away from cellular transcription to sites of viral transcription could have a significant impact on genome instability in KSHV infected cells. An additional consideration is how the induction of DNA damage during the early stages of KSHV lytic replication could lead to mutations in progeny cells during cancer development. It is established that early events in primary KSHV infection of target cells involves the expression of a subset of lytic and latent viral genes prior to the establishment of latency [71]. This includes genes that require the activity of ORF57 for their expression, including K8, K8.1 and ORF59 [72], [73]. It seems likely then that expression of ORF57 during *de novo* infection prior to the onset of latency could give rise to a background level of genome instability in infected cells, as has been suggested previously [17].

Interestingly, recent data suggests that KSHV lytic replication leads to genome instability, although no underlying mechanism was described [17]. This is in addition to observations of chromosomal abnormalities in KSHV infected cells and KSHV-associated tumours [18]–[24]. Furthermore, it has been suggested that the DNA damage checkpoint response could function as an anticancer barrier in KSHV infected cells, as KSHV v-cyclin expression leads to a DNA damage response [69]. Importantly, other herpesviruses are also known to lead to a DSB response [74]–[76]. This could have significant implications, as all herpesviruses encode an ORF57 homologue that interacts with the cellular mRNA export machinery [65], [67], [77]–[79]. However, not all herpesviruses are associated with cancer development. Therefore, the R-loop induced DNA damage could be a side-effect of virus replication, although with persistent infections where lytic replication plays a role in tumourigenesis, as is the case with KSHV, this could be an important driving force behind mutation and cancer development. Alternatively, the DNA damage observed in this study caused by ICP27 during HSV-1 lytic replication may have less impact during the viral cycle where persistent infection is not linked to tumourigenesis.

Interestingly, the genome instability caused by the formation of R-loops by ORF57-mediated sequestration of hTREX does not fully explain the observation that ORF57 can induce chromosome instability in the form of chromosome lagging. This observation is, however, intimately linked to the sequestration of hTREX. Depletion of UAP56, as well as other components of hTREX, by siRNA knockdown has been shown to lead to a large increase in chromosomal instability [31]. Loss of UAP56 is shown to lead to premature sister chromatid separation which is known to be a cause of micronuclei formation [80]. This could have significant implications for cells deficient in UAP56 and hTREX because of the strong link between micronuclei formation, genome instability and cancer [7], [81]. Importantly, both chromosome lagging and micronuclei formation have been reported previously in KSHV infected cells [18]. Therefore, loss of functional hTREX due to sequestration by KSHV ORF57 may cause genome instability through multiple mechanisms including the induction of R-loops as presented here, but also through aberrations in mitosis that can lead to micronuclei formation, work that is currently being investigated in our laboratory.

In summary, our data sheds new light on the mechanism of R-loop induced genome instability in the oncogenic herpesvirus, KSHV. We have demonstrated that sequestration of hTREX to sites of viral transcription by the KSHV ORF57 protein leads to severe DNA damage, and that R-loop formation is the cause of this genetic instability. We have shown the importance of the hTREX complex in maintaining genome instability in KSHV infected cells, and that loss of function could be a significant cause of double strand breaks. Importantly, when combined with data that shows loss of hTREX components in multiple cancers this model could be an important mechanism of DNA damage during tumorigenesis. Our work highlights a novel mechanism by which KSHV can induce genome instability in infected cells, an enabling characteristic of the hallmarks of cancer. It also demonstrates the importance of further understanding the complex links between mRNA processing and genome instability and the roles that mRNA processing factors have in cancer formation and progression.

## Materials and Methods

### Reagents and cell lines

pmCherry-N1, pEGFP-C1 and pEGFP-N1 were obtained from Clontech. pmCherry-ORF57 was cloned into pmCherry-N1, and pEGFP-ORF57 and pORF57pmut were cloned into pEGFP-N1, all described previously [47], [49], [61]. pMSCVgfp::AID contains full-length human AID and was obtained from Addgene (Addgene plasmid 15925) [64]. pEGFP-RNH contains full-length human RNAse H1 gene and was a kind gift from Dr Anneloor ten Asbroek [82]. EGFP-ICP27 contains the ICP27 gene from HSV-1 in the pEGFP-N1 vector, and the EGFP-ICP27 WRL mutant contains ICP27 with the point mutations W105A R107A L108A [67]. Etoposide was purchased from Cambridge Bioscience and cell treatments were as described in the text.

For the inducible iORF57-293 cell line, ORF57 was cloned into pcDNA5/FRT/TO and produced using the Flp-In T-REx system (Life Technologies) following the manufacturer's instructions and as previously described [83]. Both the Flp-In T-REx parental cell line and the iORF57-293 cell line were grown in DMEM supplemented with 10% v/v foetal bovine serum and penicillin/streptomycin (Lonza). The TREx BCBL Rta cell line was provided by Professor Jae Jung [51] and grown in RPMI supplemented with 10% foetal bovine serum and penicillin/streptomycin. Both cell lines were kept under hygromycin B selection at a concentration of 100 µg/ml, and inductions of both cell lines were performed using 2 µg/ml doxycyclin. The HEK-293T cell line was obtained from the Health Protection Agency Culture Collection and grown in DMEM supplemented with 10% foetal bovine serum and penicillin/streptomycin. The 293 rKSHV.219 cells are a 293T cell line containing a recombinant bacterial artificial chromosome harbouring the KSHV genome [60]. Cells were frown in DMEM supplemented with 10% foetal bovine serum and penicillin/streptomycin. Transfections were performed using Lipofectamine 2000 (Life Technologies), as previously described [84].

The monoclonal antibodies to GAPDH and fibrillarin and the polyclonal antibodies to RFP and lamin B1 were purchased from Abcam and used at 1∶5000, 1∶1000, 1∶1000 and 1∶2500, respectively. The monoclonal antibodies to Myc and Flag and the polyclonal antibody to Flag were from Sigma and were used at 1∶2500, 1∶1000 and 1∶1000, respectively for western blotting. Polyclonal Flag was used at 1∶250 for immunofluorescence. Monoclonal ORF57 antibody was obtained from Santa Cruz and used at 1∶1000 for western blotting and 1∶100 for immunofluorescence, and the monoclonal antibody to γH2A.x and polyclonal antibody to H2A.x were from BioLegend and used at 1∶2500 and 1∶1000, respectively for western blotting. Monoclonal γH2A.x was used at 1∶100 for immunofluorescence. Secondary antibodies for western blotting were HRP-conjugated polyclonal goat anti-mouse and polyclonal goat anti-rabbit supplied by Dako. Fluorescently-conjugated secondary antibodies were all obtained from Life Technologies and used at 1∶500: monoclonal Alexa Fluor 488, monoclonal Alexa Fluor 546, polyclonal Alexa Fluor 546 and polyclonal Alexa Fluor 546.

### Western blotting

Western blots were performed as previously described [85]. Briefly, protein samples were run on 10–12% polyacrylamide gels and transferred to nitrocellulose membranes via semi-dry blotting. Membranes were blocked with TBS + 0.1% v/v Tween 20 and 5% w/v dried skimmed milk powder. Membranes were probed with relevant primary and secondary antibodies, treated with EZ-ECL (Geneflow), and exposed to Amersham hyperfilm ECL (GE Healthcare).

### SILAC proteomics

Full details of the SILAC proteomics methods can be found in supplementary information (Text S1). The pathway analysis was performed with the Ingenuity Systems software packet, IPA 9.0 (Ingenuity Systems, Inc).

### Comet assays

Comet assays were performed using the CometAssay ES II system (Trevigen) and performed to the manufacturer's instructions. In brief, cells were transfected as necessary and grown for 48 hours before being harvested and kept on ice in PBS. Cells were counted and diluted to 1×10^5^/ml in PBS and mixed with low melting point agarose at 37°C at a ratio of 10∶1, agarose:cells. 50 µl of the cell/agarose suspension was spread onto a 2-well CometSlide and allowed to set before being placed in lysis solution at 4°C for 1–2 hours in the dark. For the alkaline assay, slides were then immersed in alkaline unwinding solution for 20 minutes at room temperature. Electrophoresis was performed in a CometAssay ES II unit in alkaline electrophoresis buffer for 30 minutes at 21 V and subsequently washed twice in water and once in ethanol for 5 minutes each before being dried at 37°C. For the neutral assay, after lysis slides were incubated in neutral electrophoresis buffer for 30 minutes at 4°C. Electrophoresis was performed in a CometAssay ES II unit in neutral electrophoresis buffer for 45 minutes at 21 V. Slides were then incubated in DNA precipitation buffer for 30 minutes at room temperature followed by 30 minutes in 70% ethanol at room temperature before being dried at 37°C. For both neutral and alkaline assays, slides were then stained with SYBR Gold for 30 minutes and subsequently imaged on a Zeiss LSM 700 laser scanning confocal microscope. Images were exported from Zen 2011 and comets were scored using TriTek CometScore.

### Microscopy

Cell fixation and staining was performed as previously described [86], [87]. Briefly, cells were grown on sterilised glass coverslips and either transfected or induced. After a specified time cells were washed in PBS and fixed in PBS containing 4% v/v paraformaldehyde for 10 minutes, washed further in PBS and permeabilised using PBS containing 1% Triton X-100 for 10 minutes. Coverslips were then incubated with appropriate primary and secondary antibodies for 1 hour each at 37°C before being mounted onto microscope slides using Vectashield with DAPI. Slides were visualised on a Zeiss LSM 700 laser scanning confocal microscope and images analysed using Zen 2011 (Zeiss). For analysis of cell transfection by fluorescence microscopy, cells were visualised using a Leica DC 300F fluorescence microscope.

### Flow cytometry

Cells were either untreated or treated with 50 µM etoposide for 30 minutes at 37°C immediately prior to fixation. Cells were fixes in PBS containing 1% paraformaldehyde for 5 minutes at room temperature and washed twice in PBS. Cells (2×10^6^ cells/ml) were permeabilised in 1× permeabilisation buffer (PBS with 1.25 mM EDTA, 2% foetal calf serum, 0.5% Triton X-100) for 20 minutes on ice before being incubated on ice for 1 hour with primary antibody. Cells were washed in PBS for 10 minutes before being incubated in secondary antibody for 1 hour on ice and washed again for 10 minutes in PBS. Fluorescence was measured using the 288 nm laser of a Becton Dickson BD-LSRFortessa flow cytometer and the proportion of fluorescent cells was calculated using the DiVa 6 software, as previously described [88].

### Fluorescence *in situ* hybridisation

To detect polyadenylated RNA, an HPLC-purified oligo dT(70) probe labelled at the 5′ end with Alexa Fluor 546 NHS Ester was used. 293T cells were attached to coverslips coated with poly-L-lysine and transfected for 24 hours before being washed once with PBS and fixed for 10 minutes in PBS with 4% paraformaldehyde, washed three times in PBS, permeabilised in PBS with 0.5% Triton X-100 for 5 minutes, washed twice more with PBS and once with 2× SSC for 10 minutes, all at room temperature. The oligo dT(70) probe was then added at 1 ng/µl in ULTRAbyb-oligo hybridisation buffer (Ambion) for 16 hours at 42°C. Cells were washed for 15 minutes each, twice with 2× SSC, once with 1× SSC and once with PBS all at room temperature. Coverslips were mounted onto microscope slides using Vectashield with DAPI. Slides were visualised on a Zeiss LSM 700 laser scanning confocal microscope and images analysed using Zen 2011 (Zeiss).

## Supporting Information

Figure S1 **Preparation of samples for SILAC analysis.** iORF57-293 cells were grown in DMEM R0K0 and induced for 24 hours, or grown in DMEM R6K4 and uninduced. (**A**) Cells were fractionated to allow for analysis of the whole cell proteome in different cellular compartments. (**B**) Cell lysates were analysed by western blotting using an antibody against FLAG to detect ORF57 expression. Fractionation was confirmed using monoclonal antibodies to endogenous GAPDH and fibrillarin, and polyclonal antibody to lamin B1.
(TIF)

Figure S2 **293 Flp-In cells show γH2A.x foci upon treatment with etoposide.** (**A**) 293 Flp-In cells were grown on poly-L-lysine coated coverslips and left either untreated or treated with 50 µM etoposide for 30 minutes. A monoclonal antibody against γH2A.x and secondary monoclonal Alexa Fluor 546 was used to demonstrate cells with DSBs, which were visualised using confocal microscopy. (**B**) 293 Flp-In cells were left either untreated or treated with 50 µM etoposide for 30 minutes. Cells were then fixed and treated with a monoclonal antibody against γH2A.x and secondary monoclonal Alexa Fluor 546 and were analysed by flow cytometry to demonstrate the number of cells with γH2A.x in untreated (**i**) or treated with etoposide (**ii**) conditions.
(TIF)

Figure S3 **Protein levels of ORF57 and Aly in iORF57-293 cells, transfected HEK 293T cells and 293T rKSHV.219 cells.** (**A**) iORF57-293 cells were either left uninduced or induced for 16 hours and cells were harvested and lysed. (**B**) HEK 293T cells were either mock transfected or transfected with EGFP-ORF57 for 24 hours and cells harvested and lysed. (**C**) 293T rKSHV.219 cells were either left unreactivated or reactivated using 20 ng/ml TPA and 1.5 mM sodium butyrate for 36 hours, cells harvested and lysed. Western blotting was carried out on all samples to look at protein levels using monoclonal antibodies to ORF57, GAPDH and Aly.
(TIF)

Figure S4 **FISH analysis of polyadenylated RNA in cells expressing EGFP-ORF57 shows a retention of cellular mRNA.** HEK 293T cells were either mock transfected, transfected with EGFP or transfected with EGFP-ORF57. FISH analysis was performed with an oligo dT(70) probe to detect polyadenylated RNA and confocal microscopy performed to visualise cells. Merged images show the red and green channels only for polyadenylated RNA and EGFP.
(TIF)

Figure S5 **Comet assays of cells mock transfected, transfected with pMSCVgfp::AID or transfected with EGFP-RNaseH1.** (**A**) HEK 293T cells were either mock transfected or transfected with pMSCVgfp::AID or EGFP-RNaseH1 for 24 hours and alkaline comet assays were performed. (**B**) Comet tails were scored using CometScore and n- and *P*-values are represented for all data and error bars show the standard error from the mean.
(TIF)

Table S1 **The five top pathway hits from SILAC analysis of iORF57-293 cells.** Fold-increases of proteins from the top pathway hits when using the Ingenuity Systems software packet, IPA 9.0 (Ingenuity Systems, Inc.).
(PDF)

Text S1 **SILAC proteomics materials and methods.**
(PDF)

## References

[1] Hanahan D, Weinberg RA (2011) Hallmarks of Cancer: The Next Generation. Cell 144: 646–674. 21376230 10.1016/j.cell.2011.02.013

[2] Hanahan D, Weinberg RA (2000) The Hallmarks of Cancer. Cell 100: 57–70. 10647931 10.1016/s0092-8674(00)81683-9

[3] Friedberg EC (2003) DNA damage and repair. Nature 421: 436–440. 12540918 10.1038/nature01408

[4] Fleck O, Nielsen O (2004) DNA repair. J Cell Sci 117: 515–517. 14730007 10.1242/jcs.00952

[5] Ishino Y, Nishino T, Morikawa K (2006) Mechanisms of Maintaining Genetic Stability by Homologous Recombination. Chem Rev 106: 324–339. 16464008 10.1021/cr0404803

[6] Jackson SP (2002) Sensing and repairing DNA double-strand breaks. Carcinogenesis 23: 687–696. 12016139 10.1093/carcin/23.5.687

[7] Crasta K, Ganem NJ, Dagher R, Lantermann AB, Ivanova EV, et al (2012) DNA breaks and chromosome pulverization from errors in mitosis. Nature 482: 53–58. 22258507 10.1038/nature10802 PMC3271137

[8] Stephens PJ, Greenman CD, Fu B, Yang F, Bignell GR, et al (2011) Massive Genomic Rearrangement Acquired in a Single Catastrophic Event during Cancer Development. Cell 144: 27–40. 21215367 10.1016/j.cell.2010.11.055 PMC3065307

[9] Forment JV, Kaidi A, Jackson SP (2012) Chromothripsis and cancer: causes and consequences of chromosome shattering. Nat Rev Cancer 12: 663–670. 22972457 10.1038/nrc3352

[10] Thompson LH, Schild D (2002) Recombinational DNA repair and human disease. Mutat Res 509: 49–78. 12427531 10.1016/s0027-5107(02)00224-5

[11] Elliott B, Jasin M (2002) Human Genome and Diseases: Double-strand breaks and translocations in cancer. Cell Mol Life Sci 59: 373–385. 11915950 10.1007/s00018-002-8429-3 PMC11146114

[12] Thompson S, Compton D (2011) Chromosomes and cancer cells. Chromosome Res 19: 433–444. 21190130 10.1007/s10577-010-9179-y PMC3770937

[13] Ganem D (2010) KSHV and the pathogenesis of Kaposi sarcoma: listening to human biology and medicine. J Clin Invest 120: 939–949. 20364091 10.1172/JCI40567 PMC2847423

[14] Chang Y, Cesarman E, Pessin MS, Lee F, Culpepper J, et al (1994) Identification of herpesvirus-like DNA sequences in AIDS-associated Kaposi's sarcoma. Science 266: 1865–1869. 7997879 10.1126/science.7997879

[15] Ye F, Lei X, Gao SJ (2011) Mechanisms of Kaposi's Sarcoma-Associated Herpesvirus Latency and Reactivation. Adv Virol 2011 Article ID 193860. 10.1155/2011/193860 PMC3103228 21625290

[16] Ganem D (2006) KSHV infection and the pathogenesis of Kaposi's sarcoma. Ann Rev Pathol Mech Dis 1: 273. 10.1146/annurev.pathol.1.110304.100133 18039116

[17] Xiao Y, Chen J, Liao Q, Wu Y, Peng C, et al (2013) Lytic infection of Kaposi's Sarcoma-Associated Herpesvirus induces DNA double-strand breaks and impairs NHEJ. J Gen Virol 94: 1870–1875. 23677788 10.1099/vir.0.053033-0

[18] Pan H, Zhou F, Gao S-J (2004) Kaposi's Sarcoma-Associated Herpesvirus Induction of Chromosome Instability in Primary Human Endothelial Cells. Cancer Res 64: 4064–4068. 15205312 10.1158/0008-5472.CAN-04-0657 PMC5257260

[19] Pyakurel P, Pak F, Mwakigonja A, Kaaya E, Biberfeld P (2007) KSHV/HHV-8 and HIV infection in Kaposi's sarcoma development. Infect Agent Cancer 2: 4. 17270056 10.1186/1750-9378-2-4 PMC1800836

[20] Popescu N, Zimonjic D, Leventon-Kriss S, Bryant J, Lunardi-Iskandar Y, et al (1996) Deletion and translocation involving chromosome 3 (p14) in two tumorigenic Kaposi's sarcoma cell lines. J Natl Cancer Inst 88: 450–455. 8618237 10.1093/jnci/88.7.450

[21] Casalone R, Albini A, Righi R, Granata P, Toniolo A (2001) Nonrandom chromosome changes in Kaposi sarcoma: cytogenetic and FISH results in a new cell line (KS-IMM) and literature review. Cancer Genet Cytogenet 124: 16–19. 11165317 10.1016/s0165-4608(00)00241-7

[22] Pyakurel P, Montag U, Castanos-Velez E, Kaaya E, Christensson B, et al (2006) CGH of microdissected Kaposi's sarcoma lesions reveals recurrent loss of chromosome Y in early and additional chromosomal changes in late tumor stages. AIDS 20: 1805–1812. 16954721 10.1097/01.aids.0000244199.72887.3d

[23] Kiuru-Kuhlefelt S, Sarlomo-Rikala M, Larramendy M, Soderlund M, Hedman K, et al (2000) FGF4 and INT2 oncogenes are amplified and expressed in Kaposi's sarcoma. Mod Pathol 13: 433–437. 10786811 10.1038/modpathol.3880074

[24] Nair P, Pan H, Stallings RL, Gao S-J (2006) Recurrent genomic imbalances in primary effusion lymphomas. Cancer Genet Cytogenet 171: 119–121. 17116491 10.1016/j.cancergencyto.2006.07.003 PMC2799290

[25] Siddiqui N, Borden KLB (2012) mRNA export and cancer. Wiley Interdiscip Rev RNA 3: 13–25. 21796793 10.1002/wrna.101

[26] Reed R, Cheng H (2005) TREX, SR proteins and export of mRNA. Curr Opin Cell Biol 17: 269–273. 15901496 10.1016/j.ceb.2005.04.011

[27] Luna R, Rondón AG, Aguilera A (2012) New clues to understand the role of THO and other functionally related factors in mRNP biogenesis. Biochim Biophys Acta 1819: 514–520. 22207203 10.1016/j.bbagrm.2011.11.012

[28] Schumann S, Jackson BR, Baquero-Perez B, Whitehouse A (2013) Kaposi's sarcoma-associated herpesvirus ORF57 protein: exploiting all stages of viral mRNA processing. Viruses 5: 1901–1923. 23896747 10.3390/v5081901 PMC3761232

[29] Culjkovic-Kraljacic B, Borden KLB (2013) Aiding and abetting cancer: mRNA export and the nuclear pore. Trends Cell Biol 23: 328–335. 23582887 10.1016/j.tcb.2013.03.004 PMC3700650

[30] Dominguez-Sanchez M, Saez C, Japon M, Aguilera A, Luna R (2011) Differential expression of THOC1 and ALY mRNP biogenesis/export factors in human cancers. BMC Cancer 11: 77. 21329510 10.1186/1471-2407-11-77 PMC3050854

[31] Yamazaki T, Fujiwara N, Yukinaga H, Ebisuya M, Shiki T, et al (2010) The Closely Related RNA helicases, UAP56 and URH49, Preferentially Form Distinct mRNA Export Machineries and Coordinately Regulate Mitotic Progression. Mol Biol Cell 21: 2953–2965. 20573985 10.1091/mbc.E09-10-0913 PMC2921121

[32] Huertas P, Aguilera A (2003) Cotranscriptionally Formed DNA:RNA Hybrids Mediate Transcription Elongation Impairment and Transcription-Associated Recombination. Mol Cell 12: 711–721. 14527416 10.1016/j.molcel.2003.08.010

[33] Dominguez-Sanchez MS, Barroso S, Gomez-Gonzalez B, Luna R, Aguilera A (2011) Genome instability and transcription elongation impairment in human cells depleted of THO/TREX. PLoS Genet 7: e1002386. 22144908 10.1371/journal.pgen.1002386 PMC3228816

[34] Jackson BR, Noerenberg M, Whitehouse A (2012) The Kaposi's sarcoma-associated herpesvirus ORF57 protein and its multiple roles in mRNA biogenesis. Frontiers Micro 3: 59. 10.3389/fmicb.2012.00059 PMC3282479 22363332

[35] Sandri-Goldin RM (2008) The many roles of the regulatory protein ICP27 during herpes simplex virus infection. Front Biosci 13: 5241–5256. 18508584 10.2741/3078

[36] Toth Z, Stamminger T (2008) The human cytomegalovirus regulatory protein UL69 and its effect on mRNA export. Front Biosci 13: 2939–2949. 17981767 10.2741/2899

[37] Goodwin DJ, Hall KT, Giles MS, Calderwood MA, Markham AF, et al (2000) The carboxy terminus of the herpesvirus saimiri ORF 57 gene contains domains that are required for transactivation and transrepression. J Gen Virol 81: 2253–2265. 10950983 10.1099/0022-1317-81-9-2253

[38] Malik P, Blackbourn DJ, Cheng MF, Hayward GS, Clements JB (2004) Functional co-operation between the Kaposi's sarcoma-associated herpesvirus ORF57 and ORF50 regulatory proteins. J Gen Virol 85: 2155–2166. 15269354 10.1099/vir.0.79784-0

[39] Palmeri D, Spadavecchia S, Carroll KD, Lukac DM (2007) Promoter- and cell-specific transcriptional transactivation by the Kaposi's sarcoma-associated herpesvirus ORF57/Mta protein. J Virol 81: 13299–13314. 17913801 10.1128/JVI.00732-07 PMC2168867

[40] Majerciak V, Yamanegi K, Allemand E, Kruhlak M, Krainer AR, et al (2008) Kaposi's sarcoma-associated herpesvirus ORF57 functions as a viral splicing factor and promotes expression of intron-containing viral lytic genes in spliceosome-mediated RNA splicing. J Virol 82: 2792–2801. 18184716 10.1128/JVI.01856-07 PMC2258979

[41] Boyne JR, Jackson BR, Taylor A, Macnab SA, Whitehouse A (2010) Kaposi's sarcoma-associated herpesvirus ORF57 protein interacts with PYM to enhance translation of viral intronless mRNAs. Embo J 29: 1851–1864. 20436455 10.1038/emboj.2010.77 PMC2885933

[42] Boyne JR, Jackson BR, Whitehouse A (2010) ORF57: Master regulator of KSHV mRNA biogenesis. Cell Cycle 9: 2702–2703. 20686356 10.4161/cc.9.14.12627

[43] Boyne JR, Whitehouse A (2006) gamma-2 Herpes virus post-transcriptional gene regulation. Clin Microbiol Infect 12: 110–117. 16441447 10.1111/j.1469-0691.2005.01317.x

[44] Massimelli MJ, Kang J-G, Majerciak V, Le S-Y, Liewehr D, et al (2011) Stability of a long noncoding viral RNA depends on a 9-nt core element at the RNA 5′ end to interact with viral ORF57 and cellular PABPC1. Int J Biol Sci 7: 1145–1160. 22043172 10.7150/ijbs.7.1145 PMC3204405

[45] Massimelli MJ, Majerciak V, Kruhlak M, Zheng Z-M (2013) Interplay between Polyadenylate-Binding Protein 1 and Kaposi's Sarcoma-Associated Herpesvirus ORF57 in Accumulation of Polyadenylated Nuclear RNA, a Viral Long Noncoding RNA. J Virol 87: 243–256. 23077296 10.1128/JVI.01693-12 PMC3536381

[46] Sei E, Conrad NK (2011) Delineation of a core RNA element required for Kaposi's sarcoma-associated herpesvirus ORF57 binding and activity. Virology 419: 107–116. 21889182 10.1016/j.virol.2011.08.006 PMC3177971

[47] Jackson BR, Boyne JR, Noerenberg M, Taylor A, Hautbergue GM, et al (2011) An interaction between KSHV ORF57 and UIF provides mRNA-adaptor redundancy in herpesvirus intronless mRNA export. PLoS Pathog 7: e1002138. 21814512 10.1371/journal.ppat.1002138 PMC3141038

[48] Malik P, Blackbourn DJ, Clements JB (2004) The evolutionarily conserved Kaposi's sarcoma-associated herpesvirus ORF57 protein interacts with REF protein and acts as an RNA export factor. J Biol Chem 279: 33001–33011. 15155762 10.1074/jbc.M313008200

[49] Boyne JR, Colgan KJ, Whitehouse A (2008) Recruitment of the complete hTREX complex is required for Kaposi's sarcoma-associated herpesvirus intronless mRNA nuclear export and virus replication. PLoS Pathog 4: e1000194. 18974867 10.1371/journal.ppat.1000194 PMC2569588

[50] Han Z, Swaminathan S (2006) Kaposi's sarcoma-associated herpesvirus lytic gene ORF57 is essential for infectious virion production. J Virol 80: 5251–5260. 16699005 10.1128/JVI.02570-05 PMC1472138

[51] Nakamura H, Lu M, Gwack Y, Souvlis J, Zeichner SL, et al (2003) Global Changes in Kaposi's Sarcoma-Associated Virus Gene Expression Patterns following Expression of a Tetracycline-Inducible Rta Transactivator. J Virol 77: 4205–4220. 12634378 10.1128/JVI.77.7.4205-4220.2003 PMC150665

[52] Yuan J, Adamski R, Chen J (2010) Focus on histone variant H2AX: To be or not to be. FEBS Lett 584: 3717–3724. 20493860 10.1016/j.febslet.2010.05.021 PMC3695482

[53] Majerciak V, Kruhlak M, Dagur PK, McCoy JP Jr, Zheng ZM (2010) Caspase-7 cleavage of Kaposi sarcoma-associated herpesvirus ORF57 confers a cellular function against viral lytic gene expression. J Biol Chem 285: 11297–11307. 20159985 10.1074/jbc.M109.068221 PMC2857008

[54] Jha HC, Upadhyay SK, AJ MP, Lu J, Cai Q, et al (2013) H2AX Phosphorylation Is Important for LANA-Mediated Kaposi's Sarcoma-Associated Herpesvirus Episome Persistence. J Virol 87: 5255–5269. 23449797 10.1128/JVI.03575-12 PMC3624323

[55] Ong S-E, Blagoev B, Kratchmarova I, Kristensen DB, Steen H, et al (2002) Stable Isotope Labeling by Amino Acids in Cell Culture, SILAC, as a Simple and Accurate Approach to Expression Proteomics. Mol Cell Proteomics 1: 376–386. 12118079 10.1074/mcp.m200025-mcp200

[56] Munday DC, Surtees R, Emmott E, Dove BK, Digard P, et al (2012) Using SILAC and quantitative proteomics to investigate the interactions between viral and host proteomes. Proteomics 12: 666–672. 22246955 10.1002/pmic.201100488

[57] Cox J, Mann M (2008) MaxQuant enables high peptide identification rates, individualized p.p.b.-range mass accuracies and proteome-wide protein quantification. Nat Biotech 26: 1367–1372. 10.1038/nbt.1511 19029910

[58] Cox Jr, Neuhauser N, Michalski A, Scheltema RA, Olsen JV, et al (2011) Andromeda: A Peptide Search Engine Integrated into the MaxQuant Environment. J Proteome Res 10: 1794–1805. 21254760 10.1021/pr101065j

[59] Fenech M, Kirsch-Volders M, Natarajan AT, Surralles J, Crott JW, et al (2011) Molecular mechanisms of micronucleus, nucleoplasmic bridge and nuclear bud formation in mammalian and human cells. Mutagenesis 26: 125–132. 21164193 10.1093/mutage/geq052

[60] Vieira J, O'Hearn PM (2004) Use of the red fluorescent protein as a marker of Kaposi's sarcoma-associated herpesvirus lytic gene expression. Virology 325: 225–240. 15246263 10.1016/j.virol.2004.03.049

[61] Taylor A, Jackson BR, Noerenberg M, Hughes DJ, Boyne JR, et al (2011) Mutation of a C-terminal motif affects KSHV ORF57 RNA binding, nuclear trafficking and multimerisation. J Virol 85: 7881–7891. 21593148 10.1128/JVI.00138-11 PMC3147935

[62] Gomez-Gonzalez B, Felipe-Abrio I, Aguilera A (2009) The S-phase checkpoint is required to respond to R-loops accumulated in THO mutants. Mol Cell Biol 29: 5203–5213. 19651896 10.1128/MCB.00402-09 PMC2747986

[63] Wahba L, Amon JD, Koshland D, Vuica-Ross M (2011) RNase H and Multiple RNA Biogenesis Factors Cooperate to Prevent RNA:DNA Hybrids from Generating Genome Instability. Mol Cell 44: 978–988. 22195970 10.1016/j.molcel.2011.10.017 PMC3271842

[64] Dickerson SK, Market E, Besmer E, Papavasiliou FN (2003) AID Mediates Hypermutation by Deaminating Single Stranded DNA. J Exp Med 197: 1291–1296. 12756266 10.1084/jem.20030481 PMC2193777

[65] Tian X, Devi-Rao G, Golovanov AP, Sandri-Goldin RM (2013) The Interaction of the Cellular Export Adaptor Protein Aly/REF with ICP27 Contributes to the Efficiency of Herpes Simplex Virus 1 mRNA export. J Virol 87: 7210–7217. 23637401 10.1128/JVI.00738-13 PMC3700301

[66] Volcy K, Fraser N (2013) DNA damage promotes herpes simplex virus-1 protein expression in a neuroblastoma cell line. J Neurovirol 19: 57–64. 23354549 10.1007/s13365-012-0140-z PMC3572510

[67] Tunnicliffe RB, Hautbergue GM, Kalra P, Jackson BR, Whitehouse A, et al (2011) Structural basis for the recognition of cellular mRNA export factor REF by herpes viral proteins HSV-1 ICP27 and HVS ORF57. PLoS Pathog 7: e1001244. 21253573 10.1371/journal.ppat.1001244 PMC3017119

[68] Boyne JR, Whitehouse A (2009) Nucleolar disruption impairs Kaposi's sarcoma-associated herpesvirus ORF57-mediated nuclear export of intronless viral mRNAs. FEBS Lett 583: 3549–3556. 19850040 10.1016/j.febslet.2009.10.040

[69] Koopal S, Furuhjelm JH, Järviluoma A, Jäämaa S, Pyakurel P, et al (2007) Viral Oncogene–Induced DNA Damage Response Is Activated in Kaposi Sarcoma Tumorigenesis. PLoS Pathog 3: e140. 10.1371/journal.ppat.0030140 PMC1994968 17907806

[70] Singh VV, Dutta D, Ansari MA, Dutta S, Chandran B (2014) Kaposi's Sarcoma-Associated Herpesvirus Induces the ATM and H2AX DNA Damage Response Early during De Novo Infection of Primary Endothelial Cells, Which Play Roles in Latency Establishment. J Virol 88: 2821–2834. 24352470 10.1128/JVI.03126-13 PMC3958070

[71] Chandran B (2010) Early Events in Kaposi's Sarcoma-Associated Herpesvirus Infection of Target Cells. J Virol 84: 2188–2199. 19923183 10.1128/JVI.01334-09 PMC2820927

[72] Majerciak V, Pripuzova N, McCoy JP, Gao SJ, Zheng ZM (2007) Targeted disruption of Kaposi's sarcoma-associated herpesvirus ORF57 in the viral genome is detrimental for the expression of ORF59, K8alpha, and K8.1 and the production of infectious virus. J Virol 81: 1062–1071. 17108026 10.1128/JVI.01558-06 PMC1797518

[73] Krishnan HH, Naranatt PP, Smith MS, Zeng L, Bloomer C, et al (2004) Concurrent Expression of Latent and a Limited Number of Lytic Genes with Immune Modulation and Antiapoptotic Function by Kaposi's Sarcoma-Associated Herpesvirus Early during Infection of Primary Endothelial and Fibroblast Cells and Subsequent Decline of Lytic Gene Expression. J Virol 78: 3601–3620. 15016882 10.1128/JVI.78.7.3601-3620.2004 PMC371072

[74] Shirata N, Kudoh A, Daikoku T, Tatsumi Y, Fujita M, et al (2005) Activation of Ataxia Telangiectasia-mutated DNA Damage Checkpoint Signal Transduction Elicited by Herpes Simplex Virus Infection. J Biol Chem 280: 30336–30341. 15964848 10.1074/jbc.M500976200

[75] Kudoh A, Fujita M, Zhang L, Shirata N, Daikoku T, et al (2005) Epstein-Barr Virus Lytic Replication Elicits ATM Checkpoint Signal Transduction While Providing an S-phase-like Cellular Environment. J Biol Chem 280: 8156–8163. 15611093 10.1074/jbc.M411405200

[76] Gaspar M, Shenk T (2006) Human cytomegalovirus inhibits a DNA damage response by mislocalizing checkpoint proteins. Proc Natl Acad Sci U S A 103: 2821–2826. 16477038 10.1073/pnas.0511148103 PMC1413835

[77] Lischka P, Toth Z, Thomas M, Mueller R, Stamminger T (2006) The UL69 Transactivator Protein of Human Cytomegalovirus Interacts with DEXD/H-Box RNA Helicase UAP56 To Promote Cytoplasmic Accumulation of Unspliced RNA. Mol Cell Biol 26: 1631–1643. 16478985 10.1128/MCB.26.5.1631-1643.2006 PMC1430265

[78] Hiriart E, Farjot G, Gruffat H, Nguyen MVC, Sergeant A, et al (2003) A Novel Nuclear Export Signal and a REF Interaction Domain Both Promote mRNA Export by the Epstein-Barr Virus EB2 Protein. J Biol Chem 278: 335–342. 12403791 10.1074/jbc.M208656200

[79] Boyne JR, Colgan KJ, Whitehouse A (2008) Herpesvirus saimiri ORF57: a post-transcriptional regulatory protein. Front Biosci 13: 2928–2938. 17981766 10.2741/2898

[80] Rao CV, Yang Y-M, Swamy MV, Liu T, Fang Y, et al (2005) Colonic tumorigenesis in BubR1+/–ApcMin/+ compound mutant mice is linked to premature separation of sister chromatids and enhanced genomic instability. Proc Natl Acad Sci U S A 102: 4365–4370. 15767571 10.1073/pnas.0407822102 PMC555497

[81] Hatch E M, Fischer A H, Deerinck T J, Hetzer M W (2013) Catastrophic Nuclear Envelope Collapse in Cancer Cell Micronuclei. Cell 154: 47–60. 23827674 10.1016/j.cell.2013.06.007 PMC3749778

[82] ten Asbroek ALMA, van Groenigen M, Nooij M, Baas F (2002) The involvement of human ribonucleases H1 and H2 in the variation of response of cells to antisense phosphorothioate oligonucleotides. Euro J Biochem 269: 583–592. 10.1046/j.0014-2956.2001.02686.x 11856317

[83] Griffiths DA, Abdul-Sada H, Knight LM, Jackson BR, Richards K, et al (2013) Merkel cell polyomavirus small T antigen targets the NEMO adaptor protein to disrupt inflammatory signalling. J Virol 87: 13853–13867. 24109239 10.1128/JVI.02159-13 PMC3838273

[84] Gould F, Harrison SM, Hewitt EW, Whitehouse A (2009) Kaposi's sarcoma-associated herpesvirus RTA promotes degradation of the Hey1 repressor protein through the ubiquitin proteasome pathway. J Virol 83: 6727–6738. 19369342 10.1128/JVI.00351-09 PMC2698570

[85] Goodwin DJ, Whitehouse A (2001) A gamma-2 herpesvirus nucleocytoplasmic shuttle protein interacts with importin alpha 1 and alpha 5. J Biol Chem 276: 19905–19912. 11278515 10.1074/jbc.M009513200

[86] Griffiths R, Whitehouse A (2007) Herpesvirus saimiri episomal persistence is maintained via interaction between open reading frame 73 and the cellular chromosome-associated protein MeCP2. J Virol 81: 4021–4032. 17267510 10.1128/JVI.02171-06 PMC1866103

[87] Hall KT, Giles MS, Calderwood MA, Goodwin DJ, Matthews DA, et al (2002) The Herpesvirus Saimiri Open Reading Frame 73 Gene Product Interacts with the Cellular Protein p32. J Virol 76: 11612–11622. 12388722 10.1128/JVI.76.22.11612-11622.2002 PMC136780

[88] Stevenson AJ, Clarke D, Meredith DM, Kinsey SE, Whitehouse A, et al (2000) Herpesvirus saimiri-based gene delivery vectors maintain heterologous expression throughout mouse embryonic stem cell differentiation in vitro. Gene Ther 7: 464–471. 10757019 10.1038/sj.gt.3301130

