# Supplementary figures and images for: A Novel Mechanism Inducing Genome Instability in Kaposi's Sarcoma-Associated Herpesvirus Infected Cells
Source: PLoS Pathog. 2014 May 1;10(5):e1004098. doi: 10.1371/journal.ppat.1004098 (PMC4006916; doi:10.1371/journal.ppat.1004098)

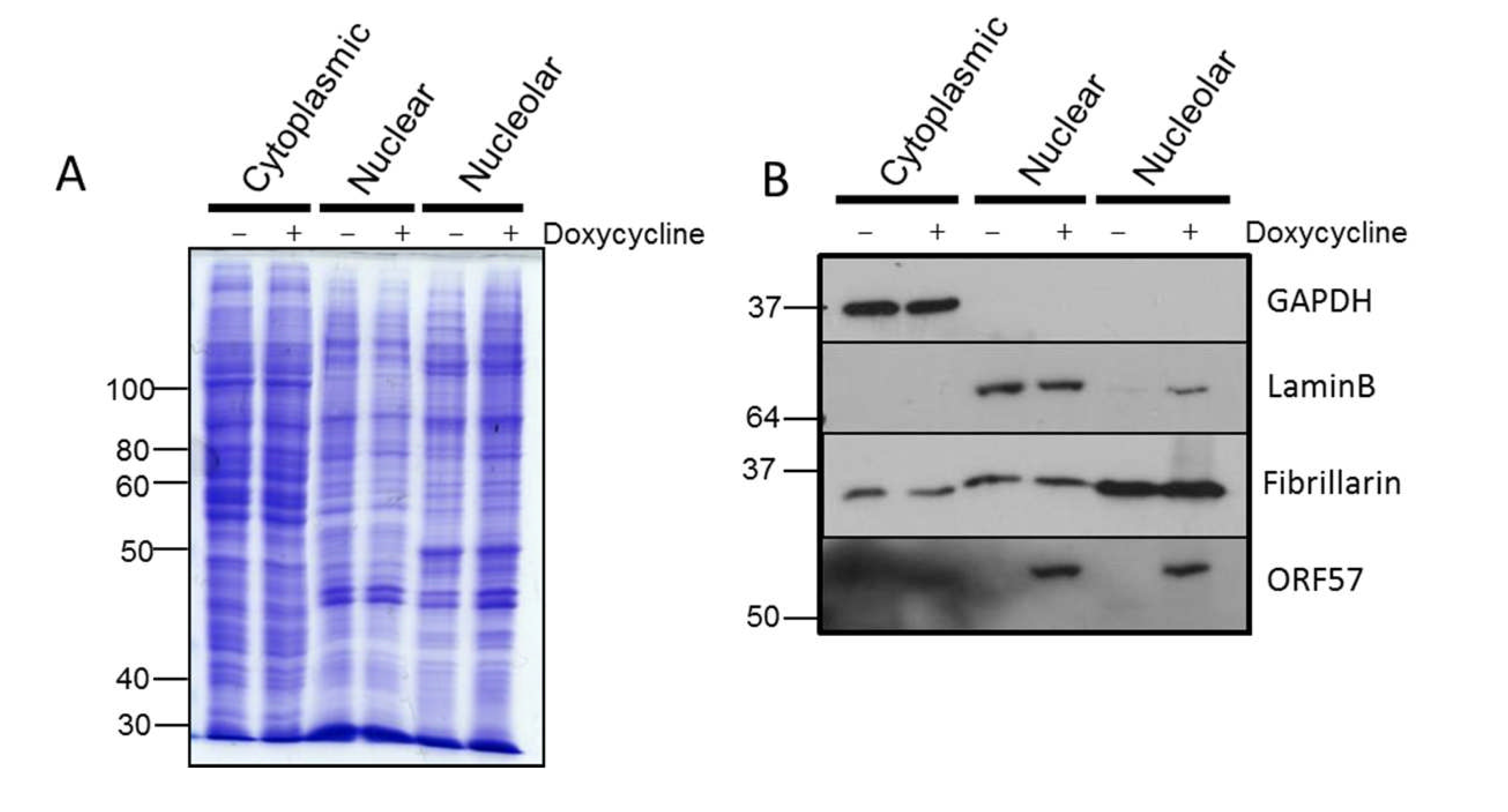

Supplement: Figure S1 — Preparation of samples for SILAC analysis. iORF57-293 cells were grown in DMEM R0K0 and induced for 24 hours, or grown in DMEM R6K4 and uninduced. (A) Cells were fractionated to allow for analysis of the whole cell proteome in different cellular compartments. (B) Cell lysates were analysed by western blotting using an antibody against FLAG to detect ORF57 expression. Fractionation was confirmed using monoclonal antibodies to endogenous GAPDH and fibrillarin, and polyclonal antibody to lamin B1. (TIF) [file ppat.1004098.s001.tif]

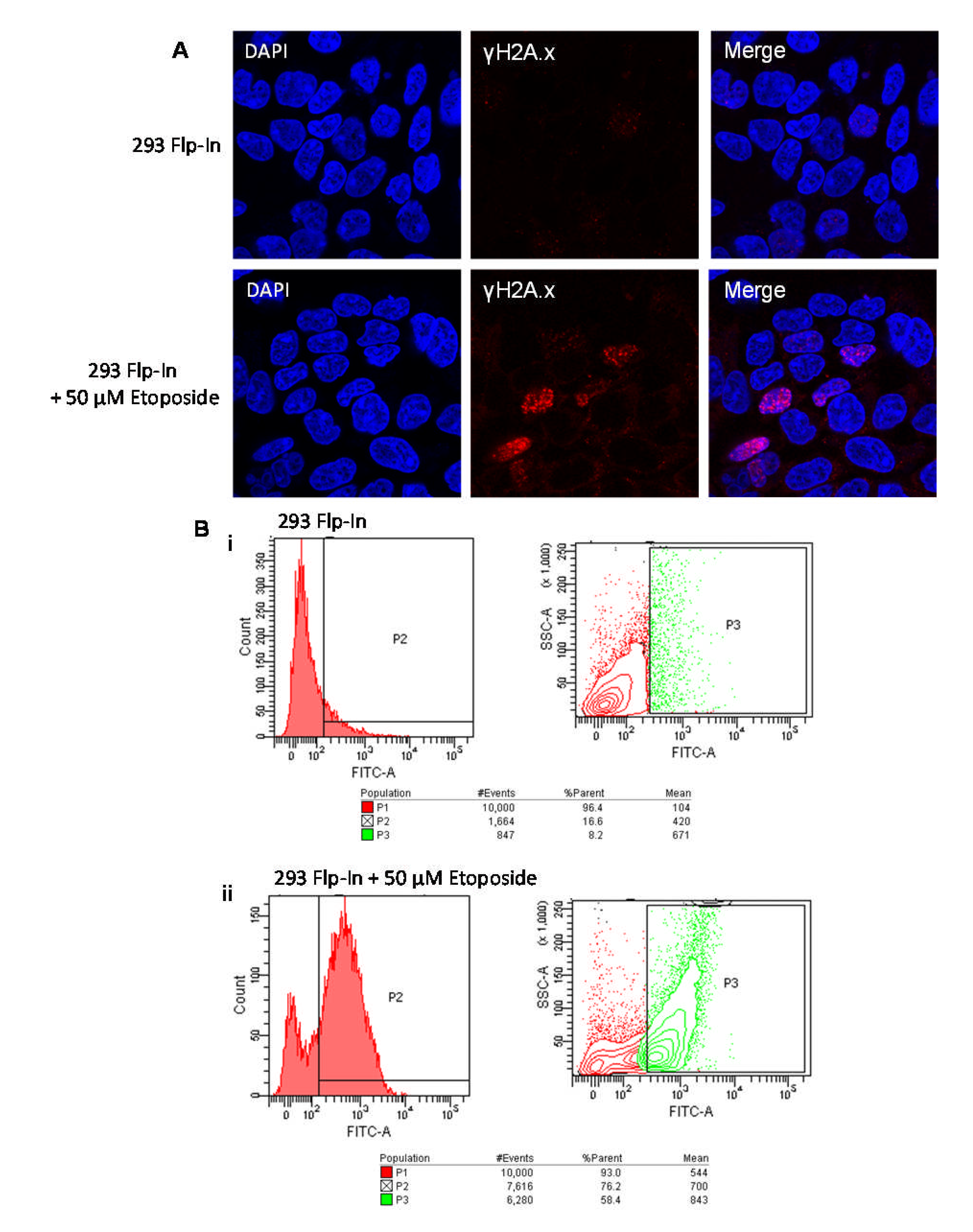

Supplement: Figure S2 — 293 Flp-In cells show γH2A.x foci upon treatment with etoposide. (A) 293 Flp-In cells were grown on poly-L-lysine coated coverslips and left either untreated or treated with 50 µM etoposide for 30 minutes. A monoclonal antibody against γH2A.x and secondary monoclonal Alexa Fluor 546 was used to demonstrate cells with DSBs, which were visualised using confocal microscopy. (B) 293 Flp-In cells were left either untreated or treated with 50 µM etoposide for 30 minutes. Cells were then fixed and treated with a monoclonal antibody against γH2A.x and secondary monoclonal Alexa Fluor 546 and were analysed by flow cytometry to demonstrate the number of cells with γH2A.x in untreated (i) or treated with etoposide (ii) conditions. (TIF) [file ppat.1004098.s002.tif]

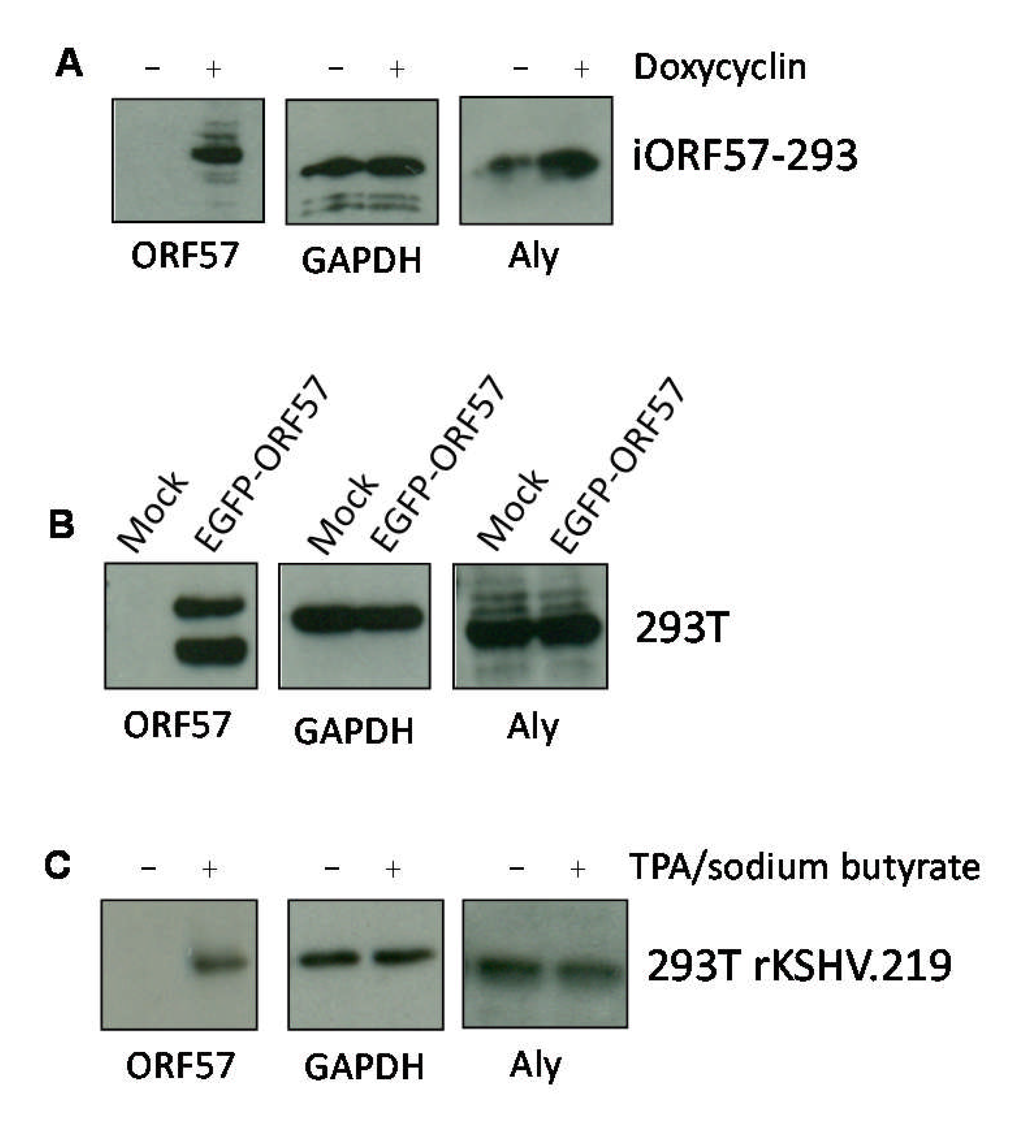

Supplement: Figure S3 — Protein levels of ORF57 and Aly in iORF57-293 cells, transfected HEK 293T cells and 293T rKSHV.219 cells. (A) iORF57-293 cells were either left uninduced or induced for 16 hours and cells were harvested and lysed. (B) HEK 293T cells were either mock transfected or transfected with EGFP-ORF57 for 24 hours and cells harvested and lysed. (C) 293T rKSHV.219 cells were either left unreactivated or reactivated using 20 ng/ml TPA and 1.5 mM sodium butyrate for 36 hours, cells harvested and lysed. Western blotting was carried out on all samples to look at protein levels using monoclonal antibodies to ORF57, GAPDH and Aly. (TIF) [file ppat.1004098.s003.tif]

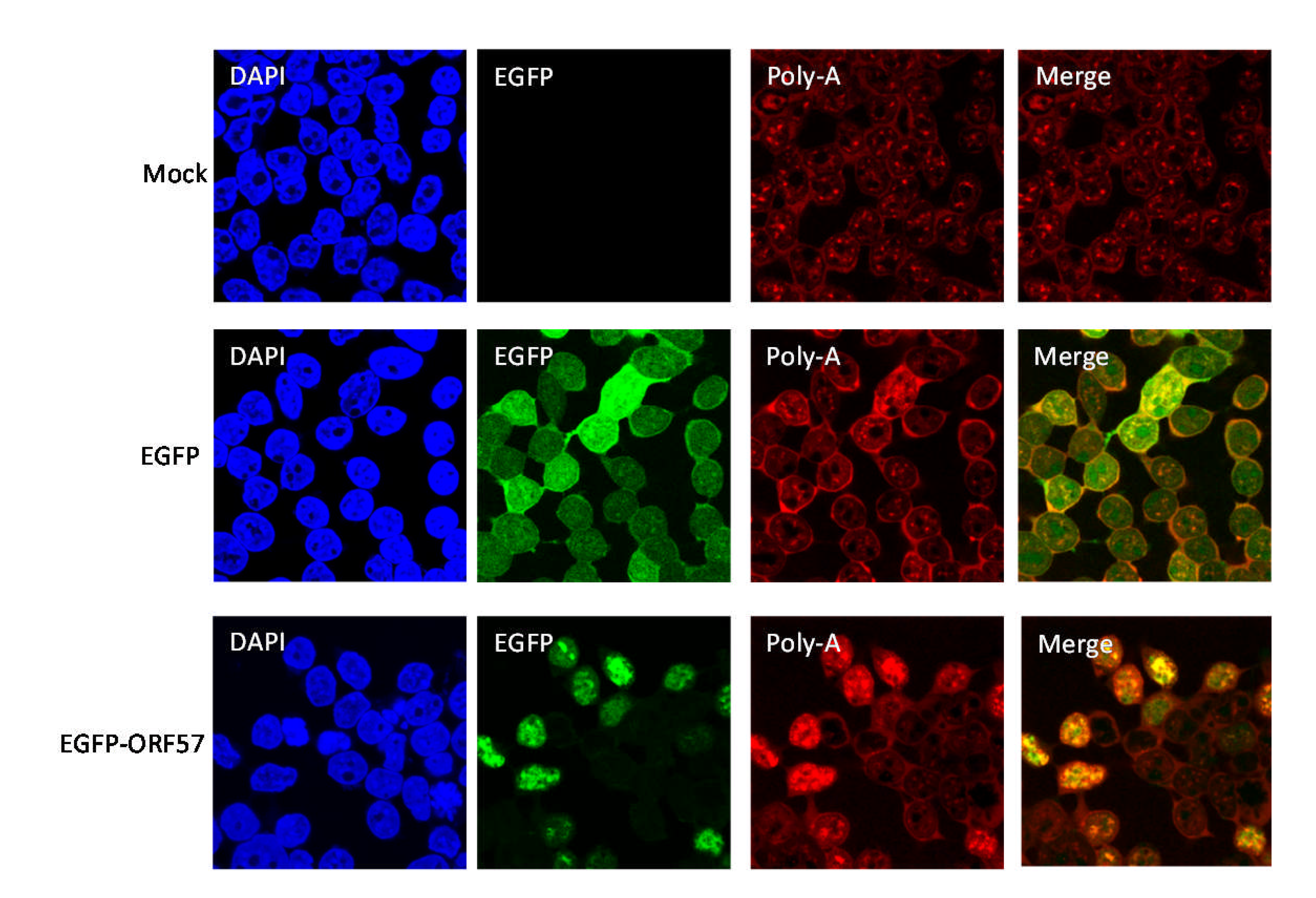

Supplement: Figure S4 — FISH analysis of polyadenylated RNA in cells expressing EGFP-ORF57 shows a retention of cellular mRNA. HEK 293T cells were either mock transfected, transfected with EGFP or transfected with EGFP-ORF57. FISH analysis was performed with an oligo dT(70) probe to detect polyadenylated RNA and confocal microscopy performed to visualise cells. Merged images show the red and green channels only for polyadenylated RNA and EGFP. (TIF) [file ppat.1004098.s004.tif]

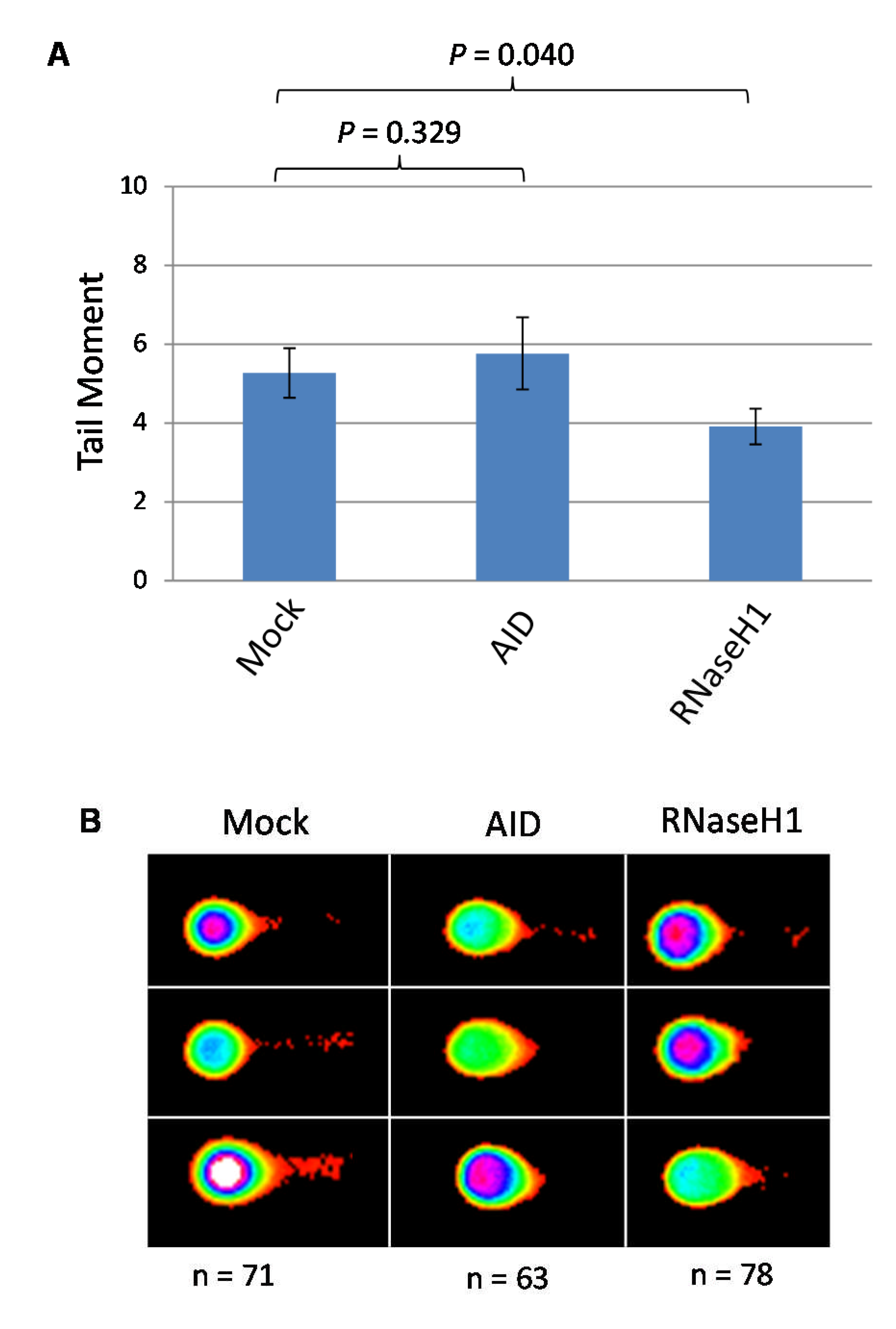

Supplement: Figure S5 — Comet assays of cells mock transfected, transfected with pMSCVgfp::AID or transfected with EGFP-RNaseH1. (A) HEK 293T cells were either mock transfected or transfected with pMSCVgfp::AID or EGFP-RNaseH1 for 24 hours and alkaline comet assays were performed. (B) Comet tails were scored using CometScore and n- and P-values are represented for all data and error bars show the standard error from the mean. (TIF) [file ppat.1004098.s005.tif]
