# Supplementary material for: A Novel Mechanism Inducing Genome Instability in Kaposi's Sarcoma-Associated Herpesvirus Infected Cells
Source: PLoS Pathog. 2014 May 1;10(5):e1004098. doi: 10.1371/journal.ppat.1004098 (PMC4006916; doi:10.1371/journal.ppat.1004098)
Supplement: Table S1 — The five top pathway hits from SILAC analysis of iORF57-293 cells. Fold-increases of proteins from the top pathway hits when using the Ingenuity Systems software packet, IPA 9.0 (Ingenuity Systems, Inc.). (PDF) [file ppat.1004098.s006.pdf]

Table S1. The five top pathway hits from SILAC analysis of iORF57-293 cells. Fold-increases of proteins from the top pathway hits when using the Ingenuity Systems software packet, IPA 9.0 (Ingenuity Systems, Inc.).

Protein	Uniprot number	Fold increase	Number of peptide hits
**RNA Post-Transcriptional Modification, Genetic Disorder, Skeletal and Muscular Disorders**			
CDC73	Q6P1J9	3.04	8
CDC5L	Q99459	3.07	25
CPSF1	Q10570	3.20	29
CYB5R3	CYB5R3	3.19	10
EMD	P50402	3.82	7
GTF2I	P78347	3.59	33
HMGA1	Q6IPL9	4.76	2
LBR	Q14739	3.64	15
LMNA	P02545	3.76	45
LMNB1	P20700	3.54	49
LMNB2	Q03252	3.35	34
NCOA5	Q9HCD5	3.04	5
PRPF8	Q6P2Q9	3.00	121
PRPF40A	O75400	3.22	24
PTPLAD1	Q9P035	3.24	10
RAD21	O60216	3.10	11
RBM25	P49756	3.56	18
SF1	Q15637	3.87	8
SF3B1	O75533	3.45	57
SF3B2	Q13435	3.17	39
SNRPD2	P62316	3.06	8
SON	P18583	3.52	30
SRRM1	Q8IYB3	3.63	9
SRRM2	Q9UQ35	3.34	42
SRSF7	Q16629	3.02	8
TMPO	P42166	4.23	26
VIM	P08670	4.45	42
**DNA Replication, Recombination, and Repair, Cellular Assembly and Organization, Gene Expression**			
APEX1	P27695	3.29	5
CHAF1A	Q13111	3.07	6
CKAP5	Q14008	3.05	62
CSDA	P16989	5.15	11
CSE1L	P55060	3.06	46
GATAD2B	Q8WXI9	3.62	5
H2AFX	P16104	3.86	5
HDLBP	Q00341	3.40	43
HIST1H1C	P16403	3.17	17
HIST1H1D	P16402	3.49	15

HIST1H1E	P10412	3.97	17
HMGB1	Q5T7C6	8.15	12
HMGB2	P26583	7.09	12
HNRNPA2B1	P22626	3.06	24
HNRNPL	P14866	3.35	26
MAZ	P56270	3.51	6
MSH2	P43246	3.24	18
MSH6	P52701	3.79	34
MTA1	Q13330	3.44	20
NUCKS1	Q9H1E3	4.77	5
RAVER1	Q8IY67	3.32	5
RECQL	P46063	3.13	16
TFCP2	Q12800	3.50	3
XPC	Q01831	4.15	3
XRCC6	P12956	3.23	38
YBX1	P67809	3.06	11
**RNA Post-Transcriptional Modification, Gene Expression, Genetic Disorder**			
C1orf77	Q9Y3Y2	3.13	4
CAND1	Q86VP6	4.51	40
CCAR1	Q8IX12	3.10	26
ELAVL1	B4DVB8	3.11	12
GNAS	Q5JWF2	0.04	4
HNRNPAB	Q99729	3.39	14
HNRNPD	Q14103	3.59	14
KHSRP	Q92945	7.61	24
KRT18	P05783	3.78	26
PABPN1	Q86U42	3.22	6
PSIP1	O75475	3.35	21
SNW1	Q13573	3.27	8
SUB1	P53999	4.85	8
TARDBP	Q13148	3.08	11
THRAP3	Q9Y2W1	3.29	41
TLN1	Q9Y490	3.11	91
UBE2I	B0QYN7	3.02	4
VAPB	O95292	3.37	4
**Cellular Assembly and Organization, RNA Post-Transcriptional Modification, Dermatological Diseases and Conditions**			
AP3B1	O00203	3.91	13
BED4	O60885	7.39	15
CCDC47	Q96A33	3.04	12
DDX41	Q9UJV9	3.94	5
DHX38	Q92620	4.14	8
ESYT1	Q9BSJ8	3.09	22
HBS1L	Q9Y450	3.58	2

HMGB2	P26583	7.09	12
LRRC59	Q96AG4	3.03	10
MAPRE1	Q15691	3.46	7
NUP155	O75694	3.28	36
PRPF31	Q8WWY3	3.06	6
RAB10	P61026	3.23	5
RPS27A	P62979	3.12	14
SRP68	Q9UHB9	3.34	6
SRSF11	Q05519	3.63	3
THRAP3	Q9Y2W1	3.29	41
ZKSCAN5	Q9Y2L8	3.45	2
**Cell Morphology, Gene Expression, RNA Post-Transcriptional Modification**			
ATXN10	Q9UBB4	3.51	5
DPF2	Q92785	3.12	3
DYNC1H1	Q14204	3.00	277
H1F0	P07305	3.40	5
H1FX	Q92522	3.69	10
HDGFRP2	Q7Z4V5	3.83	19
HIST1H1C	P16403	3.17	17
KHSRP	Q92945	7.61	24
LARP1	Q6PKG0	3.25	23
PABPN1	Q86U42	3.22	6
PPIG	Q13427	3.91	5
RBM25	P49756	3.56	18
SCAF4	O95104	3.45	9
SMU1	Q2TAY7	3.19	16
THRAP3	Q9Y2W1	3.29	41
