# Supplementary material for: A Novel Mechanism Inducing Genome Instability in Kaposi's Sarcoma-Associated Herpesvirus Infected Cells
Source: PLoS Pathog. 2014 May 1;10(5):e1004098. doi: 10.1371/journal.ppat.1004098 (PMC4006916; doi:10.1371/journal.ppat.1004098)
Supplement: Text S1 — SILAC proteomics materials and methods. (PDF) [file ppat.1004098.s007.pdf]

Text S1

SILAC proteomics materials and methods. ORF57 inducible cell line was grown in either light media (DMEM-14, Dundee cell products) or media containing the heavy isotopes of arginine and lysine (DMEM-16, Dundee cell products) supplemented with 10% SILAC dialysed FCS (DS1003, Dundee cell products). 1.5 x 10^8 cells were used for nucleolar fractionation. Cells were dounce homogenised in hypotonic buffer (10 mM HEPES, pH 7.9, 1.5 mM $MgCl_2$, 10 mM KCl, 0.5 mM DTT, 1 x Complete Protease Inhibitor cocktail). Lysate was centrifuged at 228 x g at 4°C for 5 min. Supernatant was retained as cytoplasmic fractions. The nuclear pellet was resolubilised in 3 ml buffer S1 (0.25 mM Sucrose, 1x Complete Protease Inhibitor, 1.5 mM $MgCl_2$) and layered over 3 ml buffer S2 (0.35 mM Sucrose, 1 x Complete Protease Inhibitor) and centrifuged at 1400 x g at 4°C for 5 min. The pellet was resuspended in 3 ml buffer S2 and sonicated for 10 cycles on a Soniprep 150 (MSE) at amplitude 10 µm for 10 s with 10 s gaps. The sonicated samples were layered over a cushion of buffer S3 (0.88 mM Sucrose, 1x Complete Protease Inhibitor) and centrifuged at 2,800 x g for 10 min at 4°C. The supernatant was retained as the nucleoplasmic fraction. The pellet containing the nucleoli was washed twice by resuspension in 0.5 ml of buffer S2 and centrifugation at 2,800 x g for 5 min at 4°C.

Equal amounts of protein from unlabelled and labelled samples were combined prior to protein digestion. Briefly, samples were reduced 50 mM DTT 1x NUPAGE LDS loading buffer, and then separated by one-dimensional SDS-PAGE (4-12% Bis-Tris Novex mini-gel, Life Technologies) and visualized by colloidal Coomassie staining (Novex, Life Technologies). The entire protein gel lanes were excised and cut into 10 slices each. Every gel slice was subjected to in-gel digestion with trypsin overnight at

37°C. The resulting tryptic peptides were extracted by formic acid (1%) and acetonitile, lyophilized in a speedvac and resuspended in 1% formic acid.

Trypsin-digested peptides were separated using an Ultimate 3000 RSLC (Thermo Scientific) nanoflow LC system. On average 0.5 µg was loaded with a constant flow of 5 µl/min onto an Acclaim PepMap100 nanoViper C18 trap column (100 µm inner-diameter, 2cm; Themro Scientific). After trap enrichment, peptides were eluted onto an Acclaim PepMap RSLC nanoViper, C18 column (75 µm, 15 cm; ThermoScientific) with a linear gradient of 2–40% solvent B (80% acetonitrile with 0.08% formic acid) over 65 min with a constant flow of 300 nl/min. The HPLC system was coupled to a linear ion trap Orbitrap hybrid mass spectrometer (LTQ-Orbitrap Velos, Thermo Scientific) via a nanoelectrospray ion source (Thermo Scientific). The spray voltage was set to 1.2 kV, and the temperature of the heated capillary was set to 250 °C. Full-scan MS survey spectra (m/z 335–1800) in profile mode were acquired in the Orbitrap with a resolution of 60,000 after accumulation of 1,000,000 ions. The fifteen most intense peptide ions from the preview scan in the Orbitrap were fragmented by collision-induced dissociation (normalized collision energy, 35%; activation Q, 0.250; and activation time, 10 ms) in the LTQ after the accumulation of 10,000 ions. Maximal filling times were 1,000 ms for the full scans and 150 ms for the MS/MS scans. Precursor ion charge state screening was enabled, and all unassigned charge states as well as singly charged species were rejected. The lock mass option was enabled for survey scans to improve mass accuracy. Data were acquired using the Xcalibur software.

The raw mass spectrometric data files obtained for each experiment were collated into a single quantitated data set using MaxQuant (version 1.2.2.5) and the Andromeda

search engine software . Enzyme specificity was set to that of trypsin, allowing for cleavage N-terminal to proline residues and between aspartic acid and proline residues. Other parameters used were: (i) variable modifications, methionine oxidation, protein N-acetylation, gln à pyro-glu; (ii) fixed modifications, cysteine carbamidomethylation; (iii) database: target-decoy human MaxQuant (ipi.HUMAN.v3.68); (iv) heavy labels: R6K4 and R10K8; (v) MS/MS tolerance: FTMS- 10ppm , ITMS- 0.6 Da; (vi) maximum peptide length, 6; (vii) maximum missed cleavages, 2; (viii) maximum of labelled amino acids, 3; and (ix) false discovery rate, 1%. Peptide ratios were calculated for each arginine- and/or lysine-containing peptide as the peak area of labelled arginine/lysine divided by the peak area of nonlabelled arginine/lysine for each single-scan mass spectrum. Peptide ratios for all arginine- and lysine-containing peptides sequenced for each protein were averaged. Data is normalised using 1/median ratio value for each identified protein group per labelled sample.
